# Restoration of the Tumor Suppressor Function of Y220C-Mutant p53 by Rezatapopt, a Small-Molecule Reactivator

**DOI:** 10.1158/2159-8290.CD-24-1421

**Published:** 2025-02-14

**Authors:** Anna M. Puzio-Kuter, Lizhong Xu, Mary Kate McBrayer, Romyr Dominique, Hongju H. Li, Bruce J. Fahr, Alyssa M. Brown, Amy E. Wiebesiek, Brandon M. Russo, Chris L. Mulligan, Hong Yang, Josh Battaglia, Kimberly A. Robell, Dafydd H. Thomas, Kuo-Sen Huang, Alexander Solovyov, Benjamin D. Greenbaum, Jonathan D. Oliner, Thomas W. Davis, Melissa L. Dumble, Melissa L. Johnson, Shunbin Xiong, Peirong Yang, Guillermina Lozano, Marc M. Fellous, Binh T. Vu, Alison M. Schram, Arnold J. Levine, Masha V. Poyurovsky

**Affiliations:** 1 PMV Pharmaceuticals, Inc, Princeton, New Jersey.; 2 Computational Oncology, Department of Epidemiology & Biostatistics, Memorial Sloan Kettering Cancer Center, New York, New York.; 3 Cepter Biopartners, Nutley, New Jersey.; 4 Sarah Cannon Research Institute, Nashville, Tennessee.; 5 Division of Discovery Science, Department of Genetics, The University of Texas MD Anderson Cancer Center, Houston, Texas.; 6 Memorial Sloan Kettering Cancer Center, New York, New York.; 7 Institute for Advanced Studies, Princeton, New Jersey.

## Abstract

Restoration of the tumor suppressor function of tumor-associated p53 mutants, including the Y220C substitution, has posed a significant challenge for therapeutic discovery. In this study, we describe rezatapopt (PC14586), part of a series of compounds designed to reactivate the p53 Y220C mutant. These compounds restore p53 tumor suppressor function by correcting its conformation and enabling it to bind DNA and activate downstream target genes, thus inducing antiproliferative changes in tumor cells. Our findings are supported by biochemical and structural analysis, *in vitro* and *in vivo* transcriptomics, and functional data, revealing the recovery of multiple aspects of the wild-type p53 program. These compounds demonstrate potent antitumor activity in preclinical models as single agents and in combination with immunotherapy. Currently, rezatapopt is being evaluated in a registrational phase II clinical trial for patients with advanced solid tumors harboring the *TP53* Y220C mutation.

**Significance:**

Rezatapopt is a clinical-stage compound that offers a promising treatment option for *TP53*-mutant cancers. This study details the characterization of rezatapopt and its related compounds, which can reinstate the tumor suppressor activity of the p53 Y220C mutant. These results emphasize the potential for targeting p53 mutations in cancer therapy.

## Teaser

Rezatapopt restores tumor suppressor function to p53^Y220C^ by correcting its conformation, reactivating transcriptional programs, and inducing antitumor effects in preclinical models and ongoing clinical trials.

## Introduction

The p53 tumor suppressor protein is a transcription factor that, upon cellular stress, is stabilized and activates a transcriptional program, inhibiting cell division and initiating DNA repair. If DNA damage is extensive, p53 can trigger cell death, acting as a crucial anticancer defense (1, 2). The p53 transcriptional profile is well understood and includes hundreds of genes directly and indirectly regulated by p53 (3). Key p53 target genes include *CDKN1A* (p21) for cell-cycle arrest and *MDM2*, which encodes an E3 ligase that ubiquitylates p53, creating a negative feedback loop (4, 5). In addition to the protein-coding genes, p53 also regulates noncoding RNAs, such as long noncoding RNAs (lncRNAs) and miRNAs, which play a critical role in tumor suppression (6, 7). Although some experiments suggested that p53 directly suppresses transcription to regulate cell-cycle arrest, recent studies indicate that a subset of p53 target genes indirectly regulates p53 tumor suppressor functions (8, 9). For example, p21 blocks the activity of several cyclin–CDK complexes, leading to hypophosphorylation of the tumor suppressor retinoblastoma protein or retinoblastoma-like proteins p107 (RBL1) and p130 (RBL2), resulting in the formation of the retinoblastoma-E2F complex or the DP, retinoblastoma-like, E2F4, and MuvB (DREAM) complex. Both complexes suppress the transcription of largely overlapping subsets of cell-cycle progression-associated genes (10–12). Additionally, p53 plays a critical role in antitumor immunity by recruiting immune cells and regulating cytokines (13).

The *TP53* tumor suppressor gene, which encodes the p53 protein, is mutated and functionally inactivated in approximately 50% of human cancers, whereas the p53 pathway is indirectly deactivated in most remaining tumors (14). The loss of p53 activity removes a critical regulatory mechanism that controls proliferation, DNA repair, and the ability to initiate cell death or senescence. Although other genetic alterations exist, the majority of *TP53* mutations result from a single-base alteration (missense mutation), with most residing within the DNA binding domain (DBD) of p53, which binds selectively to the p53 response element (15). The resulting p53-mutant proteins can vary in several parameters, including their relative thermodynamic stability and DNA-binding properties (16, 17). Mutant p53 proteins have become promising targets for small molecules with the potential to restore wild-type (WT) p53 functionality in tumors.

The *TP53* Y220C mutation (*TP53* c.659A>G, p.Tyr220Cys, p.Y220C) is present in approximately 1% of all solid tumors. This single amino acid substitution creates a small pocket in the p53 protein, making it thermodynamically unstable at physiologic temperatures and unable to interact with and bind to DNA effectively. The significant structural differences and defects associated with individual missense mutations hindered earlier attempts to generate mutation-agnostic activators of mutant p53, including CP-31398, PRIMA-1, MIRA-1, and APR-246 (18–21). More recent attempts to find mutant-specific p53 reactivators identified small molecules, PhiKan083 and PhiKan5196, and their derivatives, which target the Y220C-mutant p53 protein (22–25), as well as a set of covalent compounds designed to restore p53 thermal stability (26). However, these molecules are only active under specific experimental conditions and still lack the potency needed for therapeutic potential.

This article describes the preclinical characterization of a series of small molecules designed to stabilize the Y220C-mutant p53 protein and reactivate WT p53 function by selectively binding to the crevice within the protein created by the Y220C mutation. Our data demonstrate that these molecules restore the DNA-binding activity *in vitro*, initiating the full reactivation of the p53 transcriptional program and leading to *in vivo* tumor growth inhibition (TGI) in xenograft and syngeneic mouse tumor models.

In further validation of the therapeutic potential of p53 reactivation, one of the compounds described in this article, PC14586 (rezatapopt), is currently being assessed in a phase II clinical trial in patients with advanced solid tumors harboring a *TP53* Y220C mutation after showing preliminary efficacy and a manageable safety profile in a phase I clinical trial (27).

## Results

### p53-Y220C Reactivator Compounds Induced p53 DNA Binding and Increased Levels of p53 WT Conformation in Cells

**Figure 1.**
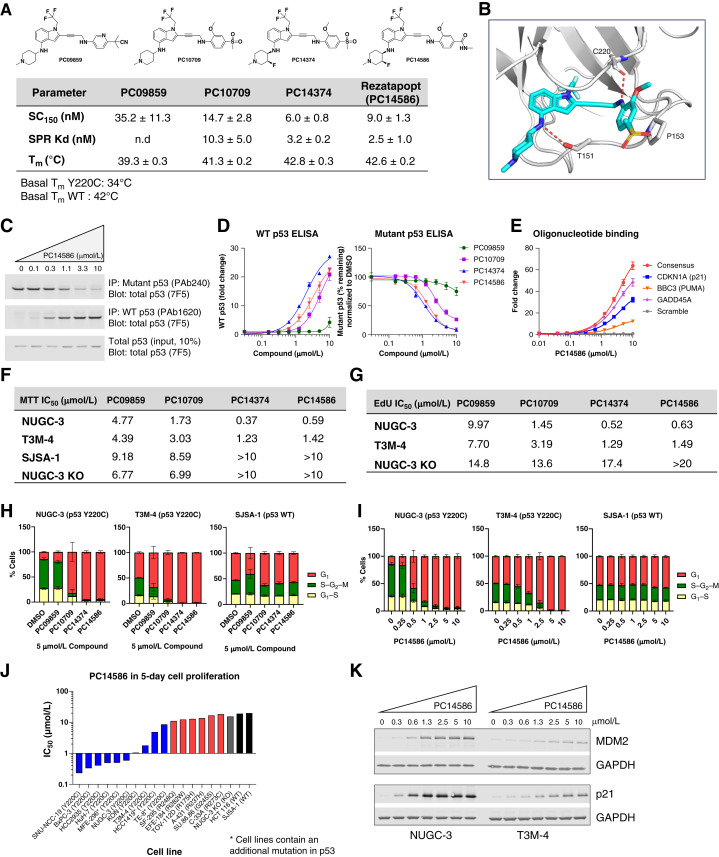
Functional characterization of p53-Y220C reactivator compounds and restoration of WT p53 function in cells. **A,** Structure of p53 reactivators and biochemical properties. The concentration of the test compound required to increase Y220C-DBD binding to the DNA from a consensus p53 response element by 1.5-fold (SC_150_) was measured by the TR-FRET assay; the binding constant (Kd) was determined by surface plasmon resonance (SPR), and the structural stabilization shown by the Tm was determined through a thermal shift assay, as described in Methods. n.d., not determined. **B,** X-ray crystal structure of the Y220C−PC10709 complex, PDB code 9BR3. The Y220C protein is shown as a gray cartoon representation with key residues highlighted as stick models. The hydrogen bonds between specific amino acids in the Y220C protein and PC10709 are indicated by dashed red lines. **C,** Induction of WT p53 antibody reactivity in cells. NUGC-3 cells were treated with PC14586 for 2 hours, followed by IP of p53 with either WT p53 (PAb1620) or mutant p53 (PAb240) cross-reacting antibodies. Changes in WT and mutant cross-reacting p53 levels were detected by Western blotting of the IP samples with the nonspecific 7F5 p53 antibody. **D,** Fold change in ELISA, normalized to the vehicle control of mutant (PAb240 antibody) and WT (PAb1620 antibody) p53 protein levels in NUGC-3 cells treated for 2 hours with increasing doses of the Y220C reactivator compounds. **E,** Biotinylated oligonucleotides corresponding to p53 response elements and a negative control (scramble) oligonucleotide were immobilized on an MSD plate and incubated with a cellular lysate from NUGC-3 cells treated with rezatapopt (PC14586; 2 hours). An increase in sequence-specific DNA binding of p53-Y220C was detected with a total p53 antibody. **F,** A 5-day MTT proliferation assay was performed as described in the Methods section using the four p53 reactivator compounds in NUGC-3 (p53-Y220C), T3M-4 (p53-Y220C), SJSA-1 (p53 WT), and NUGC-3 KO (p53 KO) cells. Proliferation rates and reactivator compound IC_50_s are presented for each cell line. **G,** The incorporation of EdU and IC_50_ was measured following a 24-hour treatment with increasing doses of the reactivator compounds in the indicated cell lines. **H,** Quantitation of the expression of fluorescently tagged proteins at specific phases of the cell cycle in NUGC-3, T3M-4, and SJSA-1 cells stably expressing the Incucyte Cell Cycle Lentivirus after a 24-hour treatment with 5 µmol/L reactivator compounds and (**I**) increasing concentrations of rezatapopt (PC14586). **J,** PC14586 IC_50_ values across various cell lines, including those harboring the *TP53* Y220C mutation or other p53 hotspot mutations, p53 KO cell lines, and p53 WT cell lines, were obtained from a 5-day MTT assay. **K,** Western blot analysis of p21 and MDM2 protein expression in NUGC-3 and T3M-4 cells following 5-hour PC14586 treatment. Data in D–I were from three experiments, data in J were from two experiments, and graphs were plotted as mean ± SD.

Small-molecule reactivators of Y220C-mutant p53 were identified through a structure-based drug design incorporating features of previously reported p53 reactivators (28). From a library of p53-Y220C reactivators, four compounds were selected for further characterization: an early hit (PC09859); a later, more potent compound (PC10709); and two of the most optimized and potent reactivators, including the clinical candidates [PC14374 and rezatapopt (PC14586)]. Structures and biochemical assessments of these four reactivators are shown in Fig. 1, with additional data provided in Supplementary Fig. S1. As the development of Y220C reactivators progressed, the ability of our compounds to induce DNA binding [measured as time-resolved fluorescence resonance energy transfer (TR-FRET) SC_150_], their binding to p53 (Kd), and the structural stabilization [melting temperature (Tm)] greatly improved (Fig. 1A; Supplementary Fig. S1A and S1B). PC14374 and PC14586 both have low nmol/L SC_150_ and SPR Kd, and the Tm of the Y220C-DBD was increased upon PC14374 and PC14586 binding, from a baseline value of ≈34°C to the melting temperature of WT p53 (≈42°C; Fig. 1A). These data suggest that our compounds stabilized the Y220C-mutant p53 protein conformation and induced sequence-specific DNA interactions, restoring WT function.

To facilitate further design, PC10709 was cocrystallized with the Y220C-DBD (Fig. 1B). The crystal structure of PC10709 complexed with the Y220C-mutant p53 protein was solved at 1.90 Å resolution. The Y220C-mutant p53 protein used for X-ray crystallization analysis consists of the DBD spanning amino acids 94 to 312, which was stabilized by four additional mutations (M133L, V203A, N239Y, and N268D) without any effect on the DNA-binding defect of the Y220C mutation (29, 30). The X-ray structure confirmed that PC10709 binds within the crevice created by the tyrosine-to-cysteine substitution, with the indole moiety occupying this space and the trifluoromethyl group extending deep into the hydrophobic pocket. The 4-aminopiperidine ring extends toward the solvent, and the alkyne linker reaches the adjacent subsite. This allows for favorable CH–π stacking interaction of the aromatic ring with Pro-153. Two critical hydrogen bonding interactions involving the p53-Y220C DBD and PC10709 were observed: one interaction with the carbonyl of Cys-220 and another with the side chain of Thr-150. Based on the analysis of the binding mode, introducing a fluorine substitution on the piperidine ring could enhance hydrogen bonding with Thr-150, increase the conformational rigidity of the piperidine ring, and reduce basicity. These modifications were incorporated into compounds PC14374 and PC14586, significantly improving potency and pharmacokinetic exposure.

The *TP53* Y220C mutation causes the mutant p53 protein to become unstable and misfolded under physiologic conditions; these distinct conformations can be monitored through changes in antibody reactivity. Specifically, PAb240, which recognizes mutant p53, and PAb1620, which recognizes correctly folded WT p53, were used for immunoprecipitation (IP) followed by Western blotting (IP-Western; refs. 31–35). The human gastric cancer cell line NUGC-3 carrying a *TP53* Y220C mutation was treated with increasing concentrations of PC14586, and a dose-dependent loss in binding to PAb240 and an increase in binding to PAb1620 were observed, whereas total p53 levels remained unchanged (Fig. 1C). The IP-Western assay was converted into an ELISA to increase throughput and sensitivity. NUGC-3 cells were treated with four compounds for 2 hours, and the effects were compared using the p53-conformation antibody ELISA. In line with the results from the biochemical assessment and IP-Western, the more potent compounds, PC14374 and PC14586, demonstrated the most robust reduction in PAb240 binding and enhancement in PAb1620 binding in NUGC-3 cells (Fig. 1D; Supplementary Fig. S1C). Additionally, ELISAs indicated that pretreating NUGC-3 cells with cycloheximide to inhibit new protein synthesis did not reduce the p53 WT conformation induced by either compound (Supplementary Fig. S1D). Combined with our *in vitro* TR-FRET results, we conclude that these compounds do not require new protein synthesis and can stabilize and reactivate existing full-length Y220C-p53 in cells. The increase in Y220C-DBD binding to p53-consensus DNA with the addition of the reactivator compounds was demonstrated in cells using an oligonucleotide pull-down assay. In line with TR-FRET results, PC14586 increased Y220-p53 binding to the indicated oligonucleotides designed from p53 response elements, including those of p53 target genes, but not to the scrambled oligonucleotide (Fig. 1E).

### p53-Y220C Reactivators Induced G_1_ Cell-Cycle Arrest in a Y220C-p53–Dependent Manner

To assess the ability of each reactivator to inhibit the growth of p53-Y220C–harboring cell lines, the four compounds were evaluated in a 5-day proliferation assay. Supporting the mechanism, cell proliferation was diminished in a dose-dependent manner following compound treatment in Y220C-mutant but not in the control [WT or p53 knockout (KO)] cells. Optimized compounds (PC14374 and PC14586), which demonstrated stronger binding in the *in vitro* assays, blocked proliferation more efficiently than earlier hits, with measured IC_50_ of 370 and 590 nmol/L in NUGC-3 cells, respectively (Fig. 1F; Supplementary Fig. S2A). Y220C-p53 selectivity was also improved with series optimization. Earlier compounds inhibited the proliferation of Y220C-harboring cells, with only 2- to 3-fold selectivity over control cells. PC14374 and PC14586 were more than 10- to 100-fold selective in the proliferation assay, thus limiting the possibility of off-target toxicities.

We obtained comparable results in the EdU (5-ethynyl-2′-deoxyuridine) incorporation assay. All compounds could block cell-cycle progression dose-dependently in the p53-Y220C cell lines but not in control cells. Lead compounds, PC14374 and PC14586, potently blocked DNA replication in NUGC-3 and T3M-4 cells with an IC_50_ between 520 nmol/L and 1.5 µmol/L (Fig. 1G; Supplementary Fig. S2A and S2B). The decrease in EdU incorporation indicates that reactivator compounds inhibited *de novo* DNA synthesis during the S-phase of the cell cycle in p53-Y220C cell lines, consistent with the upregulation of WT p53 activity. We then examined cell-cycle progression directly by live cell imaging. Following compound treatment, the extent of the induction of G_1_ cell-cycle arrest in the Y220C-expressing cells correlated with relative potency in other assays. A profound G_1_ arrest was observed in both NUGC-3 and T3M-4 cell lines treated with 5 µmol/L PC10709, PC14374, and PC14586 compared with DMSO, whereas the weaker compound PC09859 did not significantly alter the cell cycle (Fig. 1H). There was a dose-dependent increase in the accumulation of cells in G_1_ in the NUGC-3 and T3M-4 cell lines, but not in the SJSA-1 (WT p53) cell line, after treatment with PC14586 (Fig. 1I), PC14374, and PC10709 (Supplementary Fig. S2C and S2D).

The data presented thus far indicate that PC14586 is a potent and selective reactivator of p53-Y220C with strong antiproliferative effects driven by cell-cycle arrest. To further investigate the selectivity and effects on proliferation following treatment with PC14586, additional p53-Y220C, p53 hotspot mutant, and p53-WT cell lines were assessed in a 5-day 3-[4,5-dimethylthiazol-2-yl]-2,5-diphenyl tetrazolium bromide (MTT) assay. Demonstrating excellent selectivity for Y220C p53, PC14586 inhibited the proliferation of p53-Y220C cell lines with significantly lower IC_50_ values than cells harboring other p53 hotspot mutations or WT p53 (Fig. 1J; Supplementary Table S1). The less sensitive p53-Y220C cell lines, TE-8 and HCC1419, have an additional p53 mutation. In the case of TE-8, the secondary mutation, M237I, is in the DBD of p53 and has been reported to interfere with p53 DNA binding (36). The spectrum of sensitivity to our compounds in cell lines with additional p53 mutations may indicate that monitoring second-site mutations and patient responses could yield mechanistic insights and potential biomarkers.

### PC14586 Selectively Induced p53 Direct Target Gene Expression and Repressed Expression of DREAM Targets

**Figure 2.**
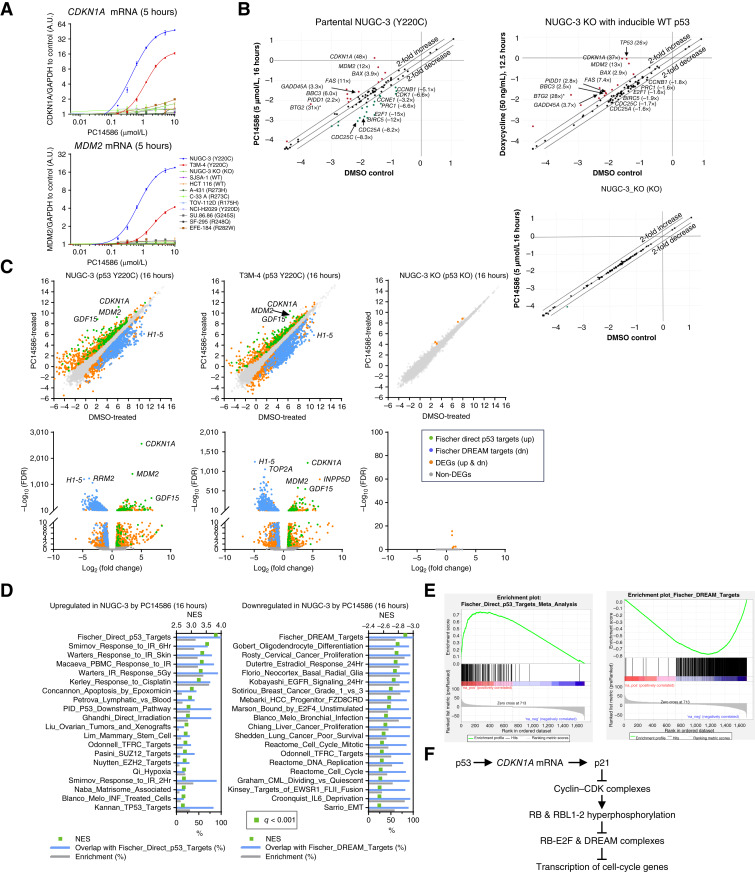
Selective restoration of WT p53 transcriptional responses by PC14586 in cells. **A,** Quantification of *CDKN1A* (p21) and *MDM2* mRNA by qRT-PCR in p53-Y220C, other p53 hotspot mutants, p53 WT, and p53 KO cell lysates following 5 hours of treatment with rezatapopt (PC14586). Data for the top four cell lines were representative results of three biological repeats, data for other cell lines were from two biological repeats, and graphs were plotted as mean ± SD. A.U., arbitrary unit. **B,** Scatter plots showing expression levels of genes involved in the p53 signaling pathway in cells following treatment with rezatapopt (PC14586). RNA samples extracted from p53-Y220C–expressing cells (NUGC-3), p53 KO cells (NUGC-3 KO) treated with rezatapopt (PC14586; 5 µmol/L, 16 hours), and NUGC-3 KO_p53i cells induced with doxycycline (50 ng/mL, 12.5 hours) were profiled by the Qiagen RT^2^ p53 pathway qRT-PCR panel. Scales are in log_10_ (relative expression to housekeeping genes; *n* = 3). **C,** Scatter plots of log_2_ (transcripts per million + 0.01) and volcano plots of −log_10_ (FDR) versus log_2_ (fold change) from RNA-seq analysis of expressed genes from p53-Y220C and p53 KO cell lines following DMSO and rezatapopt (PC14586; 5 µmol/L) treatment (16 hours). Genes enriched in gene sets of Fischer direct p53 targets (upregulated), Fischer DREAM Targets (downregulated), DEGs excluding the above-enriched genes (upregulated and downregulated), and non-DEGs were sequentially overlaid as indicated. **D,** Top enriched gene sets from GSEA of the Molecular Signatures Database C2 collection curated gene sets in the indicated RNA-seq data (NUGC-3 treated with 5 µmol/L PC14586 versus DMSO for 16 hours) from DEGs (Walden stat preranked). The number of overlapping genes from the upregulated DEGs enriched in both the Fischer direct p53 targets gene set, and each listed gene set was divided by the total number of genes in each listed gene set and then multiplied by 100 to obtain the overlap with Fischer_Direct_p53_Targets (%). Overlap with Fischer DREAM targets (%) was calculated in a similar manner, except using the downregulated DEGs. To calculate enrichment (%), the number of genes from the DEGs with the indicated direction of regulation enriched in each gene set was divided by the total number of genes in each listed gene set and multiplied by 100. Normalized enrichment scores (NES) and percentages (%) were plotted on the upper and lower *x*-axes. All gene sets shown had enrichment of FDR *q* value (*q*) <0.001. Note that the names of gene sets were shortened. See Supplementary File S11 for details. **E,** Examples of the GSEA enrichment plots from **D**. **F,** Schematic representation of p53 in the transcriptional repression of cell-cycle genes via retinoblastoma-E2F and DREAM complexes. CDK, cyclin-dependent kinase; RBL, retinoblastoma-like proteins p107 (RBL1) and p130 (RBL2).

Having observed that our reactivator compounds, including rezatapopt (PC14586), induced an accumulation of cells in the G_1_ phase and reduced the proliferation of cell lines expressing p53-Y220C, we wished to confirm that these phenotypic changes coincided with transcriptional changes associated with p53 functional activation. Indeed, a 5-hour treatment with PC14586 led to a significant increase in *CDKN1A* (p21) and *MDM2* mRNA in cells containing p53-Y220C but not in other p53-mutant, p53-WT, or p53 KO cells. The increase in mRNA expression was mirrored by the increased protein levels of p21 and MDM2 in NUGC-3 and T3M-4 cells (Fig. 1K and 2A; Supplementary Figs. S2E and S3A).

Transcriptional outcomes of Y220C-p53 reactivation were further characterized using a broader panel of 84 p53 signaling pathway–related genes. Treatment of Y220C-p53 cells (NUGC-3 and T3M-4) with PC14586 strongly induced mRNA expression of p53 direct target genes, including *CDKN1A*, *MDM2*, *BAX*, *FAS*, *GADD45A*, and *BBC3*, and downregulated p53-repressible genes regulating the cell cycle and proliferation, such as *CCNB1*, *CDK1*, *CCNE1*, *E2F1*, *BIRC5*, *CDC25A*, and *CDC25C* (Fig. 2B; Supplementary Fig. S3B; Supplementary Files S1–S9). In contrast, PC14586 exposure resulted in no significant changes in the p53-related gene expression patterns in both the WT p53 and p53 KO cell lines, demonstrating selectivity and strict dependence on the expression of the Y220C mutant for compound function. Restoration of WT p53 function was further supported by the results demonstrating that changes in the gene expression patterns in the NUGC-3 and T3M-4 cell lines treated with PC14586 were similar to or greater than those in NUGC-3 KO cells with inducible WT p53 (NUGC-3 KO_p53i; Fig. 2B; Supplementary Fig. S3B). Notably, it took longer (12.5 hours) to achieve a maximal response following doxycycline induction in the NUGC-3 KO_p53i cell line than in PC14586-treated NUGC-3 cells (5 hours). One possible explanation is that inducible p53 requires transcription and translation, whereas PC14586 can directly reactivate preexisting Y220C-mutant p53 protein in NUGC-3 cells (Supplementary Fig. S1D).

To further understand PC14586-induced gene expression changes genome-wide, the transcriptome was analyzed by next-generation sequencing (NGS) using strand-specific RNA sequencing (RNA-seq). PC14586 induced global gene expression changes in the cell lines harboring the *TP53* Y220C mutation but not in cells lacking the p53 Y220C-mutant protein (Fig. 2C; Supplementary Fig. S3C and S3D). Notably, the expression of the *CDKN1A* gene, encoding p21, was the most significantly changed, with the lowest FDRs among the upregulated genes under all conditions in both p53-Y220C cell lines (Fig. 2C; Supplementary Fig. S3C; Supplementary File S10). Gene set enrichment analysis (GSEA) showed that Fischer direct p53 targets were among the highest enriched gene sets in the upregulated differentially expressed genes (DEGs) in both NUGC-3 and T3M-4 cells (Fig. 2D and E; Supplementary Fig. S3E–S3F; Supplementary File S11). Consistent with p53 activation, other enriched gene sets, such as those related to the response to irradiation, shared many overlapping genes with the Fischer direct p53 targets gene set (Fig. 2D and E; Supplementary Fig. S3E).

Conversely, the DREAM target gene set was enriched in the downregulated DEGs (Fig. 2D and E; Supplementary Fig. S3E). Several genome-wide studies attributed p53-regulated cell-cycle arrest to the repression of the cell-cycle progression–associated genes through the p21-retinoblastoma-E2F and p21-DREAM axes, in addition to the direct upregulation of the antiproliferative genes (Fig. 2F; refs. 10–12, 37). Our transcriptome analysis indicated that PC14586 reactivation of p53 follows a similar pattern. The number of downregulated DEGs in both NUGC-3 and T3M-4 cells treated with PC14586 increased between 5 and 16 hours (Fig. 2C; Supplementary Fig. S3C), and the most enriched gene sets overlapped significantly with the Fischer DREAM targets gene set (Fig. 2D and E; Supplementary Fig. S3E). The genes most upregulated or downregulated by PC14586 in NUGC-3 and T3M-4 cells can be assigned to these two gene sets: direct p53 targets and DREAM targets (Fig. 2; Supplementary Fig. S3). Thus, our data demonstrate that PC14586 not only reactivated the p53 signaling pathway by restoring the Y220C-mutant p53 protein to WT functionality but also repressed a major pathway associated with cell-cycle progression downstream of this activation.

### Pharmacologic Activation of WT p53 in p53-Y220C Xenografts Induced p53 Signaling and Inhibited Tumor Growth

**Figure 3.**
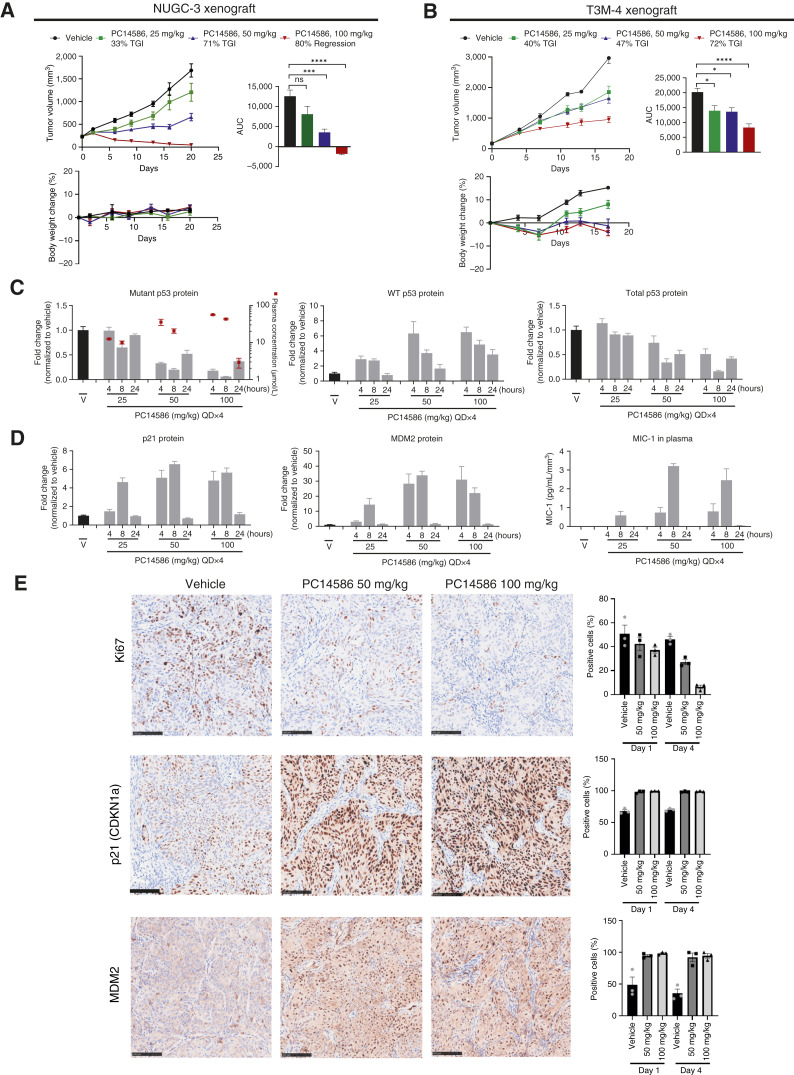
Pharmacologic activation of WT p53 in p53-Y220C xenografts induced p53 signaling and inhibited tumor growth. **A** and **B,** PC14586 was administered orally at the indicated doses to p53-Y220C–expressing (**A**) NUGC-3 and (**B**) T3M-4 mouse xenografts. Analysis includes tumor volume (mm^3^) measurements starting on day 1 of dosing (top left), AUC of tumor growth (top right), or percentage of body weight change (bottom). Each data point is the average tumor volume (left panel) or average percentage of body weight change (right). Percentage of TGI or regression is relative to starting volume. *n* = 10/group. Data shown are mean ± SEM. **C,** Fold change normalized to vehicle (V) control of mutant (PAb240 antibody), WT (PAb1620 antibody), and total (PAb1801 antibody) p53 protein levels using MSD in NUGC-3 xenograft tumors. Indicated time points are after the dose on day 4 of daily oral administration of vehicle at 25, 50, and 100 mg/kg PC14586. Consolidated vehicles from 7, 24, and 48 hours of 2QD × 1 treatments, *n* = 12; PC14586, *n* = 4/group. Data shown are mean ± SEM. Detectable plasma levels (μmol/L) are shown by the red symbol and designated on the right *y-*axis. **D,** Fold change in protein expression of p53 downstream targets using MSD (p21, MDM2) and normalized to vehicle control or ELISA (MIC-1) and normalized to vehicle control and tumor volume (mm^3^) in tumor samples described in **C**. Data shown are mean ± SEM. **E,** Representative IHC images of Ki-67, p21, and MDM2 in the NUGC-3 xenograft model after 4 days of vehicle at 50 or 100 mg/kg PC14586 daily administration. Scale bars are 100 µm, 20× objective. Bar graphs represent the average percentage of positive stained cells on days 1 and 4 following the administration of 50 and 100 mg/kg PC14586. Symbols represent individual samples. Error bars show SEM. Statistical significance relative to vehicle was determined using one-way ANOVA with Dunnett’s multiple comparisons test (adjusted *P* value * < 0.01; *** 0.0001; **** <0.0001). QD, once daily.

Functional activation following the pharmacologic restoration of WT p53 activity *in vitro* was further explored *in vivo*. PC14586 was evaluated for antitumor activity in xenografts harboring the *TP53* Y220C mutation. In the NUGC-3 xenograft model, PC14586, orally administered daily for 3 weeks (once daily × 21), demonstrated robust dose-dependent antitumor efficacy at 25 and 50 mg/kg and regression at 100 mg/kg by study end (Fig. 3A). PC14586 was well tolerated, with minimal changes in body weight and no clinical observations of toxicity across all doses (Fig. 3A). Similarly, oral daily (once daily) dosing of 25, 50, and 100 mg/kg of PC14586 resulted in 40%, 47%, and 72% TGI, respectively, and was well tolerated in the T3M-4 xenograft model (Fig. 3B). PC14586 was also administered on a bolus dosing schedule at 150 and 300 mg/kg twice daily on day 1 of a weekly dosing schedule, resulting in 92% TGI and 48% tumor regression, respectively, by day 21 in the NUGC-3 model (Supplementary Fig. S4A).

After 4 days of 25, 50, and 100 mg/kg PC14586 in the NUGC-3 xenograft model, plasma concentrations of PC14586 reached steady state and were roughly dose-proportional at 4 and 8 hours. Mutant Y220C p53 protein levels decreased at the 8-hour postdose mark with diminished binding to PAb240 at all doses, especially at 100 mg/kg, correlating to plasma concentrations of ≈42 µmol/L (Fig. 3C). Conversion of mutant p53 protein led to a dose-proportional increase in WT p53 conformation detected as a shift to increased binding to PAb1620, which decreased to baseline levels when the plasma concentration of PC14586 decreased below ≈3 µmol/L (Fig. 3C). Total p53 levels declined over time downstream of MDM2 expression. Dose-dependent reductions of mutant p53 and increases in WT p53 protein conformation at peak plasma levels were also detected in the T3M-4 xenograft model (Supplementary Fig. S4B). Although there is some variability in the overall extent of change based on cell line and the extent of MDM2 activation, total p53 levels decreased in most experimental settings. These changes suggest that the combination with MDM2 inhibitors could provide an additional boost to the activity of our compounds. However, the poor tolerability of MDM2 inhibitors could hinder the clinical translation of such a combination.

Activation of p53 downstream targets was then assessed in the tumors of the NUGC-3 xenograft model at 8 hours after dose on day 4 with increases in the p21 and MDM2 protein levels (Fig. 3D). MIC-1, encoded by another p53 target gene, *GDF15*, is a protein made in the tumor and secreted into the blood that can be measured in the plasma of mice (normalized to tumor volume) as a circulating biomarker. Similar to the other target protein levels, MIC-1 protein in the plasma increased (Fig. 3D). As conformational stabilization of the p53 protein occurs immediately following PC14586 administration and correlates with peak plasma concentrations, we tested whether markers of cell proliferation and death could also be detected immediately following drug administration. On days 1 and 4 after dosing initiation, concurrent with the increased staining for p21 and MDM2, we observed a dose- and time-dependent reduction of Ki-67 staining, indicating significant inhibition of cell proliferation (Fig. 3E). Nonsignificant increases were observed in cleaved caspase-3 and terminal deoxynucleotidyl transferase–mediated dUTP nick end labeling (TUNEL) staining (Supplementary Fig. S4C), suggesting that the inhibition of cell proliferation, rather than apoptosis, accounts for the tumor regression.

In summary, the administration of PC14586 in two xenograft models, NUGC-3 and T3M-4, resulted in dose-proportional plasma exposures leading to the rapid restoration of WT p53 function and activation of downstream targets, as shown by the increase in p21, MDM2, and MIC-1 protein levels. In line with the *in vitro* cell-cycle data, these results suggest that the administration of PC14586 blocked tumor cell proliferation in a dose- and time-dependent manner.

### PC14586 Restored Multiple Facets of the WT p53 Transcriptional Program *In Vivo*

**Figure 4.**
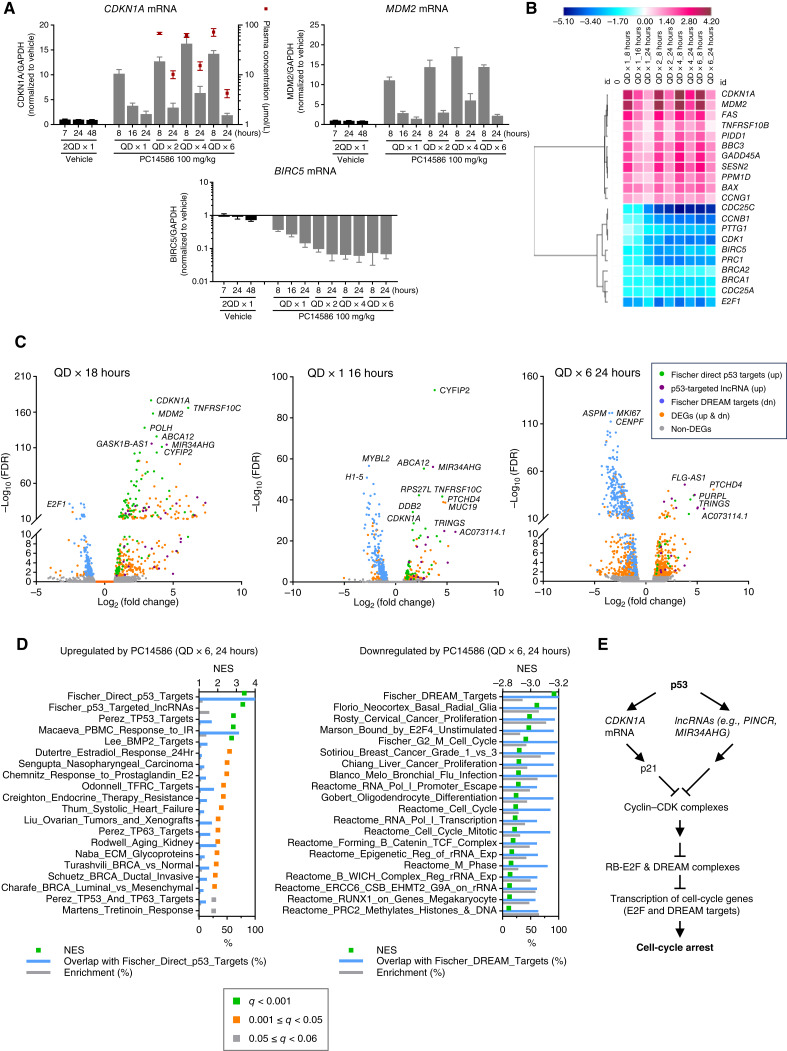
PC14586 restored multiple facets of dynamic WT p53 transcriptional responses *in vivo*. **A,** Time-dependent effects of PC14586 on gene expression of key p53 targets, *CDKN1A*, *MDM2*, and *BIRC5*, with repeat daily administration across 6 days in the NUGC-3 xenograft model. *n* = 4/group. Error bars show the SEM. The red symbols show plasma concentrations (µmol/L) and are designated on the right *y*-axis. **B,** Assessment of a panel of 84 p53 pathway genes measured by qRT-PCR. A representative number of upregulated and downregulated p53 target genes are shown across 6 days of 100 mg/kg PC14586 administration, with the mean value of log_2_ (fold change) at each time point shown on the heatmap. See Supplementary File S12 for details. RNA samples extracted from tumor samples, as in **A**, were profiled by the Qiagen RT^2^ p53 pathway qRT-PCR panel as described in the Methods section. Symbols represent the mean log_2_ (fold change) for each. **C,** Volcano plots from RNA-seq analysis of expressed genes from NUGC-3 xenograft tumors following PC14586 (100 mg/kg) and vehicle treatment as indicated. Graphs were plotted as in Fig. 2C, except that the p53-targeted lncRNA (upregulated) gene set was overlaid on top of the others. See Supplementary Fig. S5B for the corresponding scatter plots. In **B** and **C,** vehicle (consolidated), *n* = 12 and PC14586, *n* = 4 for each group, as in Figs. 3C and D and 4A. **D,** Top enriched gene sets from GSEA of the Molecular Signatures Database C2 collection curated gene sets supplemented with Fischer p53-targeted lncRNA (86 genes; C2+) in the indicated RNA-seq data [rezatapopt (PC14586) for once daily (QD) ×6 at 24 hours] from DEGs. See Supplementary File S14 for details. Gene sets with a normalized enrichment score (NES) and an FDR *q* value (*q*) < 0.001 are highlighted in green, 0.001 to 0.05 are highlighted in orange, and 0.05 to 0.06 are highlighted in gray. Graphs were plotted as in Fig. 2D. **E,** Updated schematic representation of p53 in regulating the cell cycle via retinoblastoma-E2F and DREAM complexes. Updated from Fig. 2F, the lncRNA component was added to complement p21 in regulating the cell cycle. PINCR, p53-induced noncoding RNA.

To gain further insight into the transcriptional consequences of p53 reactivation *in vivo*, we analyzed gene expression of two upregulated p53 targets, *CDKN1A* and *MDM2*, and a downregulated target, *BIRC5*, across 6 days of daily PC14586 dosing in the NUGC-3 xenografts (Supplementary Fig. S5A). Administration of PC14586 at 100 mg/kg increased *CDKN1A* and *MDM2* mRNA with peak expression at 8 hours on days 2, 4, and 6 corresponding to a plasma concentration of ≈37 µmol/L, which returned to baseline at 24 hours. *BIRC5* mRNA levels decreased without returning to baseline over time (Fig. 4A).

As with the cell-based experiments, a larger panel of genes was examined to understand temporal gene expression changes *in vivo*. Expression levels of representative upregulated and downregulated genes are shown in Fig. 4B and Supplementary File S12. The two mRNAs exhibiting the most significant and highest-fold increases were p21 (*CDKN1A*) and *MDM2* at 8 hours after the dose. Decreases in mRNA expression of selected genes were observed at 24 hours after the first dose, and expression remained low over the 6 days of dosing. *CDC25C*, *PRC1*, *CDK1*, and *CCNB1* were among the most repressed genes (Fig. 4B). The oscillatory pattern of upregulated gene expression corresponded with PC14586 and WT p53 plasma levels (Supplementary Fig. S4B), whereas the sustained suppression of downregulated genes implicated a potential indirect regulatory mechanism.

Expanding our understanding of the p53 pathway reactivation, RNA-seq analysis was carried out on tumor samples. In line with cellular data (Fig. 2B and C), the genes most affected by PC14586 treatment *in vivo* were p53 direct target genes and p21-DREAM targets (Fig. 4C). Consistent with the *in vitro* data, the most significantly upregulated genes *in vivo* included *CDKN1A* at the earlier time points (up to 8 hours after PC14586 treatment). At later time points, however, the most DEGs included *CYFIP2*, *PTCHD4*, and some lncRNAs, such as *MIR34AHG*, *FLG-AS1*, and *PURPL* (Fig. 4C; Supplementary Fig. S5B and S5C; Supplementary File S13). lncRNAs, which were upregulated by PC14586 treatment over time, were shown to be involved in p53-mediated tumor suppression. Fischer and colleagues identified 86 lncRNAs as direct p53 targets from 44 published RNA-seq datasets (7), which we added to a Molecular Signatures Database C2 curated collection of gene sets to analyze the RNA-seq data. In cell culture and tumors, p53 direct targets, DREAM targets, and p53-targeted lncRNA gene sets consistently showed increased enrichment scores or ranks (Fig. 4D and E; Supplementary Figs. S3F and S5D–S5F; Supplementary File S14). To summarize these changes, we generated a heatmap of the most upregulated and downregulated genes for PC14586 treatments. We found that these three gene sets—p53 direct targets, p53 DREAM targets, and p53 lncRNA targets—covered most of the top regulated genes (Supplementary Fig. S5G). These data illustrate that p53 activation, which resulted in the upregulation of coding and noncoding RNAs and subsequent repression of the DREAM targets, may constitute the primary mechanism of PC14586-inhibited cell and tumor growth.

In summary, PC14586 inhibited tumor growth *in vivo* by inducing WT p53 transcriptional signatures, including sustained repression of cell-cycle genes. To investigate p53’s role in the antitumor immune response, we explored the consequences of p53-Y220C reactivation in an immunocompetent mouse tumor model.

### Antitumor Activity of PC14374 in an Immunocompetent p53-Y220C (Hupki) Model

Human p53 knock-in (Hupki) mice were generated by replacing the mouse *Trp53* exons 4 to 9 with the corresponding human *TP53* exons 4 to 9 and incorporating the Y220C mutation. The Hupki mice homozygous for the Y220C mutation (Y220C/Y220C; C/C) succumbed to lymphomas and sarcomas within 6 months, whereas heterozygous mice (Y220C/+) developed tumors with a later latency and median survival time (Supplementary Fig. S6A). These data are in line with previously characterized germline p53 mutants (38). Primary cell lines were generated from tumors that arose, and five of the sarcoma cell lines were subsequently tested with PC14374 in a 5-day MTT cell proliferation assay with IC_50_ values ranging from 0.12 to 0.18 µmol/L (Supplementary Fig. S6B).

**Figure 5.**
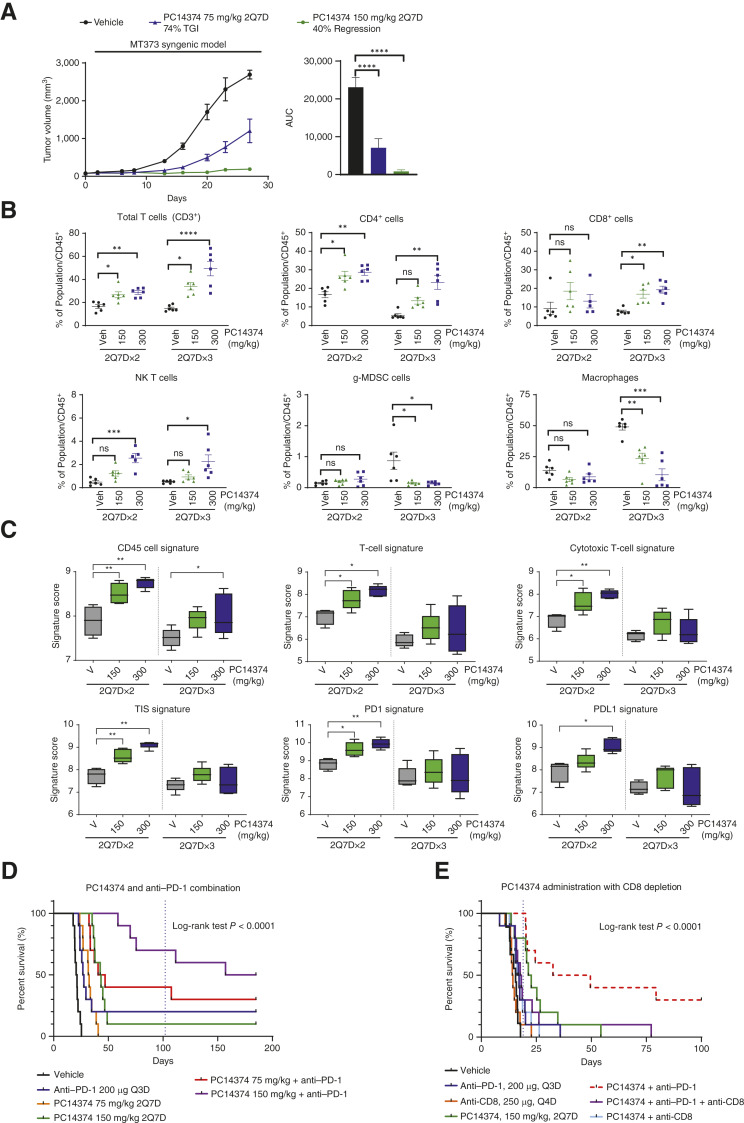
Pharmacologic activation of WT p53 inhibited tumor growth and induced immunologic cell changes in tumors. **A,** Average tumor volume (mm^3^; left), AUC analysis of tumor growth (middle), and percentage of body weight change (right) in C57Bl/6 mice bearing subcutaneous MT373 tumors treated with vehicle (V) or PC14374 at 150 or 300 mg/kg (2Q7D: two doses on day 1 only on a weekly dosing cycle). *n* =10/group. Data shown are mean ± SEM. Percentage of TGI (% TGI) or tumor regression is relative to tumor volume at study start. Statistical significance relative to the vehicle was determined using one-way ANOVA with Dunnett’s multiple comparisons tests (adjusted *P* value: **** <0.0001). **B** and **C,** Six days after inoculation with MT373 cells, mice were administered PC14374 orally at 150 or 300 mg/kg (2Q7D × 2 or 2Q7D × 3: two doses on day 1 only of a weekly dosing cycle for 2 or 3 weeks, respectively). **B,** Tumors were harvested 72 hours after last dose to analyze tumor-infiltrating lymphocytes by flow cytometry. Percentage of cell population change is normalized to CD45^+^ cells. Vehicle, *n* = 3/group; PC14374, *n* = 4/group. Error bars show SEM. Statistical significance relative to the vehicle was determined using one-way ANOVA with Dunnett’s multiple comparisons tests (adjusted *P* value: * ≤0.01; *** ≤0.001; **** ≤0.0001). **C,** Tumors harvested 72 hours after last dose were analyzed on the NanoString IO 360 panel for gene expression. Box plots represent fold change in the signature scores and the raw *P* value for the comparison between treatments for each cell signature (*, *P* ≤0.01; **, *P* ≤0.001). Vehicle control is treated as the baseline of the comparison. Vehicle, *n* = 3/group; PC14374, *n* = 4/group. Error bars show SEM. **D,** Mice bearing MT373 tumors were administered 75 mg/kg or 150 mg/kg PC14374 (2Q7D: two doses on day 1 only of a weekly dosing cycle), 200 µg anti–PD-1 (Q3D: dose every three days), or a combination of PC14374 and anti–PD-1 starting on day 6 after cell implantation. Tumor volume (mm^3^) measurements started on day 1 of dosing. Kaplan–Meier curves are shown as percentage of survival with *n* = 10/group. The dashed vertical line represents dosing discontinuation at day 102. **E,** Mice bearing MT373 tumors were administered 150 mg/kg of PC14374 (2Q7D), 200 µg of anti–PD-1 (Bio X Cell Clone RMPI-14; Q3D), or 250 µg of anti-CD8 (Bio X Cell Clone 2.43) as single agents or in combination starting on day 6 after cell implantation. Groups dosed with anti-CD8 were pretreated 3 days prior to the study start. Tumor volume (mm^3^) measurements started on day 1 of dosing. Kaplan–Meier curves are shown as percentage of survival with *n* = 10/group. The dashed vertical line represents dosing discontinuation on day 19. Statistical significance across all groups was determined using the log-rank (Mantel–Cox) test.

To evaluate PC14374 in an immune-competent setting, one of the five primary sarcoma cell lines, MT373, was implanted subcutaneously into C57Bl/6 mice. Mice bearing MT373 syngeneic tumors were administered either vehicle or PC14374 on a bolus dosing regimen of two doses on day 1 only of a weekly dosing cycle. PC14374 at 75 or 150 mg/kg twice on day 1 only of a weekly dosing cycle resulted in TGI and regression, respectively, and was well tolerated (Fig. 5A). After analyzing the pharmacodynamic response in the MT373 model at 8 hours after the dose, a reduction in mutant p53 protein levels was noted, accompanied by a corresponding increase in WT p53 protein levels at both dose levels (Supplementary Fig. S6C). The reduction of mutant p53 levels 8 hours after the dose (150 mg/kg twice on day 1 only of a weekly dosing cycle) corresponded to plasma concentrations of ≈21 µmol/L. These results suggest that PC14374 has broad activity against multiple tumor lines harboring p53-Y220C, including mouse syngeneic and human xenograft models (Figs. 3 and 5).

### p53 Restoration Resulted in Immunologic Cell Changes in Tumors and Increased Survival with Immunotherapy

It has been suggested that an essential role of p53 is modulating the immune response during cancer development. Loss of p53 function leads to the recruitment of suppressive myeloid cells through increased expression of chemokines while attenuating the CD4^+^ Th 1 and CD8^+^ T-cell responses (39). Therefore, the pharmacologic reactivation of p53 in the p53-Y220C Hupki model and tumoral immune infiltration was explored by performing immune phenotyping using a flow cytometry immune panel. Six days after inoculation with MT373 cells, C57Bl/6 mice were administered PC14374 at 150 or 300 mg/kg for two (two doses on day 1 only of a weekly dosing cycle × 2) or three (two doses on day 1 only of a weekly dosing cycle × 3) dosing cycles, with tumors being processed and analyzed for tumor-infiltrating lymphocytes by flow cytometry. Within the tumor, reactivation of p53 with PC14374 resulted in significant increases in tumor-infiltrating T cells (CD3^+^, CD4^+^, CD8^+^, and NK T cells) that were both dose- and time-dependent (Fig. 5B). PC14374 also reduced the levels of macrophages and granulocytic myeloid-derived suppressor cells (gMDSC; CD3^–^CD11b^+^Ly6c^–^Ly6g^+^) although this decrease was more prominent at the later time point (Fig. 5B).

Immunologic gene expression changes were also analyzed in tumors following p53 reactivation using the NanoString IO 360 panel (Supplementary File S15). The panel comprises 48 clinically relevant gene signatures, including the 18-gene tumor inflammation signature (TIS) and signatures associated with immune responses and the tumor microenvironment (Fig. 5C). Six days after inoculation with MT373 cells, mice received 150 or 300 mg/kg PC14374 PO twice on day 1 only of a weekly dosing cycle × 2 or twice on day 1 only of a weekly dosing cycle × 3, a similar dosing regimen to that used in the immune phenotyping of tumors. Changes in NanoString gene expression signatures following reactivation of p53 led to increases in CD45, T-cell, and cytotoxic cell signatures that are consistent with the increase in similar populations observed with flow cytometry and correlate with an immune response of functionally active NK and T cells and improved immunosurveillance. TIS, PD-1, and PD-L1 signatures associated with response to anti–PD-1/anti–PD-L1 blockage also increased (Fig. 5C).

This led to the hypothesis that p53 reactivation could combine with immune checkpoint blockade to enhance efficacy. Therefore, the Hupki syngeneic model was used to examine the consequences of coadministration of PC14374 with a PD-1 antibody. Mice were administered PC14374 at 75 or 150 mg/kg (two doses on day 1 only of a weekly dosing cycle) as single agents or with 200 µg of anti–PD-1 (every 3 days) up to 102 days, when dosing ceased. Mice were further monitored for tumor regrowth until day 185 of the study. The combination of PC14374 and anti–PD-1 proved to increase survival, with a median of 43 days observed with the single agent PC14374 (150 mg/kg), which increased to >185 days with combination treatment (Fig. 5D). The 75-mg/kg PC14374 dose in the combination treatment also improved survival, albeit to a lesser extent (median survival was 32 days with the single agent PC14374 vs. 44 days with the combination; Fig. 5D).

Given tumor infiltration of T cells after p53 reactivation, we examined the contribution of CD8^+^ T cells to the antitumor effect of PC14374 by performing CD8^+^ T-cell depletion. Three days before the start of the study, mice bearing MT373 tumors were pretreated with anti-CD8 (only groups assigned to anti-CD8 treatment) to initiate T-cell depletion. At the start of the study, mice were administered PC14374, anti–PD-1, or anti-CD8 as single agents; a combination of PC14374 with each antibody; or a combination of all three. As expected, PC14374, in combination with anti–PD-1, resulted in an increase in survival compared with the vehicle group. However, the robust antitumor activity was lost with the addition of anti-CD8 (Fig. 5E). Likewise, a similar reduction in the antitumor response was seen when PC14374 was administered with anti-CD8 compared with single-agent PC14374.

We cannot formally exclude the potential contribution of the tumor cell–autonomous changes elicited by p53 reactivation in an immunocompetent model. p53 has also been reported to transactivate key regulators in pathways involved in pathogen sensing, cytokine production, and inflammation (39). A modified analysis of the NUGC-3 tumor samples showed that PC14586 administration *in vivo* led to changes in the expression of immune and inflammatory signaling–associated genes and gene sets (*TNFSF9*, *CSF2*, *IL1A*, *GDF15*, and TNFA signaling and inflammatory response; Supplementary Fig. S6D–S6F; Supplementary File S16).

In summary, p53 reactivation led to an increase in infiltrating T cells, which suggests that CD8^+^ T cells might contribute to the antitumor effects downstream of p53 reactivation and could lead to efficacy gains with immune checkpoint blockade.

### Rezatapopt (PC14586) Treatment Resulted in Responses in the Clinical Setting in Two Heavily Pretreated Patients with Solid Tumors

**Figure 6.**
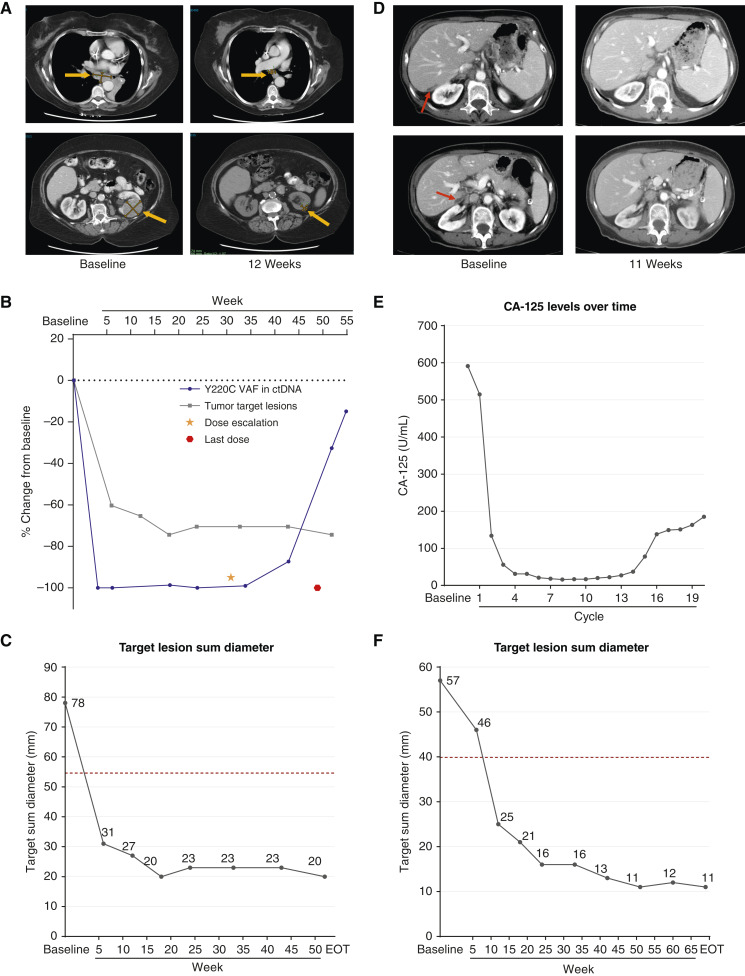
Responses of two patients with solid tumors harboring *TP53* Y220C mutations receiving rezatapopt. **A–C** Patient with advanced small cell lung carcinoma from the PYNNACLE phase I study. **A,** CT scans at baseline and 12 weeks showing a reduction in target lesions. **B,***TP53* Y220C VAF over time. **C,** Reduction in the sum diameter of target lesions over time (the dotted line represents the value that would constitute a PR; 54.6 mm). **D–F** Patient with advanced endometrial cancer from the PYNNACLE phase I study. **D,** CT scans at baseline and 11 weeks showing a reduction in target lesions. **E,** Cancer antigen 125 (CA-125) levels over time. **F,** Reduction in the sum diameter of target lesions over time (the dotted line represents the value that would constitute a PR; 39.9 mm). VAF, variant allele frequency; EOT, end of treatment.

Rezatapopt has demonstrated promising early clinical activity in patients with solid tumors harboring a *TP53* Y220C mutation (27). As a proof of concept, we describe two patients treated with rezatapopt as part of the PYNNACLE phase I dose-escalation study (NCT04585750). A patient with advanced small cell lung carcinoma had symptomatic, progressive disease after two prior lines of systemic therapy and prior radiotherapy for brain metastasis. Following the detection of a *TP53* Y220C mutation by NGS, the patient was enrolled in the PYNNACLE study and received rezatapopt 1,150 mg once daily (Supplementary Fig. S7A). After 6 weeks of treatment, the patient achieved a partial response with relief of respiratory symptoms. CT scans demonstrated target lesion reduction of 60.3% at 6 weeks and 65.4% at 12 weeks (Fig. 6A). Supporting the Y220C-p53 specific engagement and mechanism of action, treatment with rezatapopt resulted in a rapid decrease in *TP53* Y220C ctDNA, which correlated with radiographic tumor shrinkage (Fig. 6B and C). Transient treatment-related grade 3 neutropenia was observed at the 1,150 mg once-daily dose. Rezatapopt dosing was increased to 2,000 mg once daily at week 30 and was well tolerated. Treatment continued for 11 months and 28 days until disease progression of nontarget lesions.

The second patient had advanced serous endometrial cancer and was heavily pretreated with four lines of prior therapy. Disease progression and increasing cancer antigen 125 levels led to enrollment onto PYNNACLE after a *TP53* Y220C mutation was confirmed by NGS of archival tissue. The patient received rezatapopt 2,000 mg once daily (Supplementary Fig. S7B). CT scans 6 weeks into treatment revealed target lesion reduction of 17.8%, which further deepened to 55.8% at 11 weeks (Fig. 6D), and a complete response in nontarget lesions (peritoneal disease). Radiographic response was accompanied by a reduction in cancer antigen 125 levels (Fig. 6E). Unfortunately, although present in the biopsy tissue, this patient did not have detectable Y220C-p53 ctDNA at treatment initiation, and the response could not be directly correlated with changes in markers of progressive disease. Rezatapopt was well tolerated with grade 1 fatigue, fever, and asymptomatic laboratory abnormalities. After a year of receiving rezatapopt, target lesion reduction was 80.0% (Fig. 6F); however, the peritoneal disease had worsened, consistent with the progression of nontarget lesions. The patient continued to receive clinical benefit from rezatapopt and remained on therapy, completing twenty-three 3-week cycles. Treatment was discontinued after 16 months due to progressive disease. Manuscripts describing phase I data and additional clinical patients’ response data are in preparation.

Although we observed robust antitumor activity of rezatapopt in the abovementioned cases, further patient biomarker analysis and preclinical studies are needed to better understand the response mechanism in patients with complex genomic profiles. Additionally, rezatapopt had limited brain penetration in rats, and although its activity in the CNS is being evaluated, this suggests a potential direction for further reactivator discovery.

## Discussion

Our study presents the functional characterization of novel compounds that restore WT activity to the Y220C mutant p53. These reactivators, including PC14374 and PC14586, selected from a library of p53-Y220C reactivator compounds, are remarkably specific to the *TP53* Y220C mutation (28). A series of biochemical assays confirmed target-specific engagement and described the restoration of WT p53 functionality *in vitro* and in cells. The more potent reactivators (PC14374 and PC14586) inhibited p53 Y220C–carrying tumor growth in xenograft models and enhanced immune engagement in syngeneic models. Therefore, these allele-specific p53 Y220C reactivators, especially rezatapopt (PC14586), are distinct in their high selectivity and potency; more importantly, they enable clinical translation and the first investigation of Y220C-p53 reactivation in human solid tumors.

When misfolded mutant proteins aggregate in tumor cells, they are usually considered undruggable. It was postulated that in the cellular context, rescuing a thermodynamically unstable p53 mutant may require the drug to bind to p53 immediately after its biosynthesis (16). However, our data demonstrate that preexisting proteins can be reactivated in cells. We propose that an equilibrium exists between the fully misfolded aggregated state and the partially unfolded state of Y220C p53 in cells. The latter state could be protected from aggregation by yet unidentified molecular chaperones, allowing Y220C p53 to be reactivated by our compounds.

In light of our data, the potential for using small molecules to reactivate other p53 mutants is important to consider. Missense mutations in *TP53* can be categorized into several classes depending on the specific structural changes they induce. The challenge presented by most p53 mutations is that very few produce a structured pocket that could be suitable for small molecule binding. Analysis of a spectrum of p53 mutants revealed that each p53 mutation creates different subtypes of structural lesions, and each uniquely changes protein conformation; thus, tumor-derived mutants of p53 may require subtype- or mutant-selective strategies (16). Although ongoing efforts to restore function continue, rezatapopt activity against Y220C remains the only clinical validation of p53 reactivation.

Restoring WT p53 in tumors through genetic alterations or viral transduction leads to robust inhibition of growth and tumor cell death (40–42). Our selective p53-Y220C reactivators expand on these observations by converting accumulated endogenous Y220C mutant protein into the WT p53 conformation. *In vitro* and *in vivo*, p53 target genes increased rapidly, whereas downregulated genes decreased and reached maximum repression by 16 hours. Upregulation varied with plasma drug levels, indicating direct p53 regulation, whereas downregulated genes remained repressed. This suggests that p53 repression is likely indirect, downstream of directly activated genes (10, 11).

Studies have shown that p53 regulates cell-cycle arrest through the p21-retinoblastoma-E2F and p21-DREAM axes to suppress cell-cycle progression–associated genes, and our analysis of p53 reactivation by PC14586 shows a similar mechanism with p21 as the major component of cell cycle–associated gene regulation (10–12). Other genes, gene products, or pathways might also be involved. Additional top-regulated, more sustainable genes, including protein-encoding genes such as *BTG2*, *PTCHD4*, and *TNFRSF10C* and lncRNAs such as *MIR34AHG* (the host gene for miR-34a), *PINCR*, and *PURPL*, can participate in the repression of CDKs or regulate p53 activity by other mechanisms and potentially facilitate the observed repression pattern of the cell-cycle genes (7, 10–12, 37). Our description of the temporal transcriptome changes hints at the existence of multiple levels of p53-mediated gene expression regulation and provides insight into tumor suppression mechanisms used by WT p53. It is perhaps surprising that following treatment, we primarily saw changes in the transcription of cell-cycle regulators and observed little change in apoptotic markers in tumors, such as caspase-3 cleavage. We are exploring regression mechanisms in our clinical trial patient samples and preclinical studies.

*In vivo* reactivation of p53 led to changes in immune and inflammatory gene expression, likely contributing to mouse tumor regression. This resulted in increased tumor-infiltrating T cells and decreased macrophages. Reactivation of p53 also correlated with increased CD45, T-cell, and cytotoxic cell signatures, suggesting improved immunosurveillance. Increased TIS, PD-1, and PD-L1 signatures, which are associated with response to anti–PD-1/anti–PD-L1 blockade, correlated with improved survival. Although we are not yet able to correlate the observed tumor cell–autonomous changes in the transcription of immune and inflammation-related genes with the observed tumor suppression and immune component activation in the syngeneic model, our data suggest that both intrinsic and nontumor cell–autonomous factors contribute to tumor suppression following p53 reactivation.

In conclusion, our compounds, including rezatapopt, restore WT p53 function to the p53 Y220C mutant, enabling novel therapeutic strategies. Rezatapopt is being evaluated in patients with solid tumors harboring the *TP53* Y220C mutation [PYNNACLE (NCT04585750); ClinicalTrials.gov; RRID: SCR_002309; Pharma P. PYNNACLE study (cited January 15, 2025). Available from: https://www.pynnaclestudy.com/]. In the phase I portion of the trial, rezatapopt monotherapy demonstrated promising clinical activity in heavily pretreated patients across multiple tumor types, with a favorable safety profile observed within the efficacious dose range (27). The registrational phase II portion of the trial will assess rezatapopt in patients with advanced solid tumors that harbor a *TP53* Y220C mutation and are *KRAS* WT. Rezatapopt has the potential to address an unmet medical need by providing an additional therapeutic option to patients, underscoring our research’s practical implications.

## Methods

### Reagents and Equipment

RNeasy Mini Kit (Cat. # 74106), TissueLyser LT (Cat. # 85600, RRID: SCR_020428), QIAcube (Cat. # 9001292, RRID: SCR_018618), RNeasy Mini QIAcube Kit (Cat. # 74116), and RNase-Free DNase Set (Cat. # 79254) were purchased from Qiagen. The JANUS automated workstation was purchased from PerkinElmer. The BlueWasher was purchased from BlueCatBio MA Inc. The NanoDrop 2000 Spectrophotometer was purchased from Thermo Fisher Scientific (Cat. # ND-2000, RRID: SCR_018042). The LightCycler 96 (Cat. # 05815916001) and LightCycler 480 (Cat. # 05015243001, RRID: SCR_018626) were purchased from Roche. The FastLane Cell Probe Kit (Cat. # 216413), which contains cell wash buffer, FastLane Lysis Buffer, gDNA Wipeout Buffer 2, and QuantiTect Probe RT-PCR Kit, was from Qiagen. The TaqMan primer/probe sets were obtained from Applied Biosystems via Thermo Fisher Scientific: human *GAPDH* (assay ID: Hs99999905_m1, label: VIC-MGB, Cat. # 4448491), *CDKN1A* (p21; assay ID: Hs00355782_m1, label: FAM-MGB, Cat. # 4351368), *MDM2* (assay ID: Hs01066930_m1, label: FAM-MGB, Cat. # 4351368), and *BIRC5* (assay ID: Hs00153353_m1, label: FAM-MGB, Cat. # 4351368).

### TR-FRET Assay

DNA-binding activity was measured by the TR-FRET assay in which recombinant His_6_-tag Y220C p53 DBD protein binds to biotin-labeled consensus DNA, as described (28). Briefly, the binding of recombinant His_6_-tag Y220C p53 DBD protein and biotin-labeled consensus DNA is registered by the FRET between allophycocyanin conjugated to anti–His_6_ tag antibody and Europium conjugated to streptavidin. SC_150_ and SC_300_ (substrate concentrations to increase DNA binding by 1.5- and 3.0-fold, respectively) values were calculated using either Prism (GraphPad, RRID: SCR_002798) or XLfit software (IDBS).

### Thermal Shift Assay

One microliter per well of DMSO or testing compounds at different concentrations in DMSO were added to the 384-well PCR plate (HSP-3810 Bio-Rad Hard-Shell PCR plate). Eighteen microliters of 5.69 µmol/L recombinant His-tag Y220C p53 DBD protein (amino acids 94-312) in assay buffer (50 mmol/L Tris-HCl, pH 7.4; 75 mmol/L KCl; and 1 mmol/L DTT) was added. The plate was centrifuged for 1 minute at 1,200 rpm (Eppendorf centrifuge 5810R) and incubated on a plate shaker at room temperature (RT) for 10 minutes. SYPRO Orange dye (1.5 µL per well, Invitrogen, Cat. # S6650) diluted from the 300× stock in assay buffer was added. Ten microliters per well of mineral oil (Sigma) was added to prevent evaporation. The plate was centrifuged, and the assay signals were monitored by reading excitation at 465 nm and emission fluorescence at 590 nm on a FluoDia T70 reader (PTI) every 1.5°C increment from 26 to 50°C. The fluorescence intensity signals fitted to the Boltzmann equation were plotted, and the Tms were determined using GraphPad Prism software.

### Surface Plasmon Resonance

Biotinylated DBD of p53-Y220C was immobilized on each of the eight channels of a Biacore 8K instrument, achieving between 200 and 400 response units. Compounds were dissolved in DMSO and titrated over the surface. The affinity and binding kinetics were determined using a single-cycle injection technique. Compounds were diluted in 20 mmol/L HEPES-KOH (pH 7.5), 150 mmol/L KCl, 1 mmol/L DTT, 0.05% Tween 20, and 5% DMSO. Kinetics for each compound were determined with a contact time of 120 seconds, a dissociation time of 900 seconds, and a flow rate of 60 µL/minute.

### Cells

Description of cell lines, including cell sources, histologic subtype, p53 status, growth medium, cell authentication, and mycoplasma testing information, is listed in Supplementary Table S2. Cell lines were authenticated by the cell authentication service with short tandem repeat profiling at ATCC, using the FTA Sample Collection Kit for Human Cell Authentication Service (ATCC, Cat. # 135-XV-20); mycoplasma testing was performed using the Universal Mycoplasma Detection Kit (ATCC, Cat. # 30-1012K), following the manufacturer’s instructions. After purchase, each cell line was expanded and banked. Once revived or brought up from the frozen stocks, cell lines used in experiments were cultured for a maximum of 20 passages. NUGC-3 KO cell line (Japanese Collection of Research Bioresources and PMV Pharmaceuticals, generated in-house from RRID: CVCL_1612) was generated by knocking out mutant p53 from intron 1 to exon 6 using CRISPR technology. The NUGC-3 KO_p53i cell line (Japanese Collection of Research Bioresources and PMV Pharmaceuticals, generated in-house from RRID: CVCL_1612) was generated via viral transduction using the pTetOne expression vector containing WT p53 under doxycycline control (Takara Bio, Cat. # 631847). After transduction, cells were placed under puromycin selection for clonal selection. Clones were picked and screened for WT p53 expression after 24 hours of doxycycline treatment. RPMI 1640 medium (Cat. # R8758); DMEM/F-12, 1:1 mixture (Cat. # D8437); DMEM (Cat. # D5796); McCoy’s 5A (Cat. # M4892); Eagle Minimum Essential Medium (Cat. # M2279); and heat-inactivated FBS (Cat. # F8192) were purchased from Sigma-Aldrich. All cells were cultured in the indicated growth medium, supplemented with the indicated concentration of FBS in a humidified incubator with 5% CO_2_ at 37°C.

### Cell Proliferation Assay

The antiproliferative activity of compounds was evaluated using the MTT (Sigma-Aldrich, Cat. # M5655-1G) assay in a 96-well plate format. Cells were seeded at an appropriate density depending on the growth rate in 96-well microtiter plates, and compounds were tested at eight concentrations with 2-fold serial dilutions. Antiproliferative activity was assessed 5 days later by the conversion of the tetrazolium dye MTT (1 mg/mL) into insoluble formazan by viable cells, which was dissolved in ethanol and measured by a microplate reader for absorbance with a wavelength of 570 nm and a reference wavelength of 650 nm. The results were presented as a percentage of the viability of untreated cells (control). The IC_50_ was determined from the regression of a plot of the logarithm of concentration versus percentage of viability by XLfit IDBS.

### Western Blot

For each cell line, 10 µg of total protein was run on a 4% to 12% Bis-Tris precast gel or a 4% to 20% Tris-glycine precast gel (Thermo Fisher Scientific). The gel was transferred to a nitrocellulose membrane (0.45 μm pore size; Bio-Rad). The membrane was blocked for 1 hour using the Odyssey TBS blocking solution (LI-COR Biosciences). Membranes were incubated overnight in p53 (DO-1; Cell Signaling Technology, Cat. # 18032, RRID: AB_2798793), Cell Signaling or MDM2 (D1V2Z; Cell Signaling Technology, Cat. # 86934, RRID: AB_2784534), and GAPDH (14C10; Cell Signaling Technology, Cat. # 2118, RRID: AB_561053) primary antibodies. The next day, blots were washed and incubated in goat anti-mouse for p53 or goat anti-rabbit for actin secondary antibodies (IRDye 800CW goat anti-mouse IgG, Cat. # 926-32210, RRID: AB_621842; IRDye 680 goat anti-rabbit IgG, Cat. # 926-68071, RRID: AB_10956166) from LI-COR Biosciences. Blots were then washed and imaged using the Odyssey CLx imaging system (LI-COR Biosciences, Cat. # 9140-02, RRID: SCR_014579). Bands were quantified using Image Studio software (LI-COR Biosciences, RRID: SCR_015795) and normalized to the GAPDH control.

### EdU Quantification

NUGC-3, T3M-4, and NUGC-3 KO cells were plated in clear-bottom 384-well plates and allowed to adhere overnight. p53 reactivator compounds were added, and cells were incubated for 24 hours. To analyze the effects of treatment on cell growth, EdU incorporation was analyzed using Invitrogen’s Click-iT HCS assay (Thermo Fisher Scientific, Cat. # C10357). Plates were imaged on PerkinElmer’s Operetta imaging system to count and quantify the number of cells positive for EdU incorporation. The data were normalized to the DMSO control for each cell line, and the IC_50_ was determined using XLfit.

### Cell-Cycle Measurements

NUGC-3 (JCRB, Cat. # JCRB0822, RRID: CVCL_1612), T3M-4 (RIKEN BioResource Research Center, Cat. # RCB1021, RRID: CVCL_405), and SJSA-1 (ATCC, Cat. # CRL-2098, RRID: CVCL_1697) cell lines stably expressing the Incucyte Cell Cycle Lentivirus (green/orange; Sartorius, Cat. # 4809) were generated according to the manufacturers’ protocol. To determine the phase of the cell cycle after reactivator compound treatment, cells were plated in clear-bottom 96-well plates and allowed to attach for 24 hours. Cells were incubated with the compounds for 24 hours. After 24 hours, images were taken using the green, orange, and phase channels of the Incucyte. Using the basic analyzer program, the number of cells in G_1_ phase (orange), S–G2–M phase (green), and G_1_–S phase (yellow) of the cell cycle was quantified and graphed.

### Mice

Female Balb/c nude mice (RRID: IMSR_ENV:HSD-165), 4 to 6 weeks of age, were purchased from Envigo and C57Bl/6 mice (RRID: IMSR_CRL:027) were purchased from Charles River and housed at the PMV Pharmaceuticals Vivarium. Mouse care and experimental procedures were performed in accordance with all institutional guidelines for ethical use, and all research protocols were approved by the Institutional Animal Care and Use Committees of PMV Pharmaceuticals. All mice were kept in ambient temperatures of 68–72°F, 30–70% humidity, and a dark/light cycle of 12 hours.

NUGC-3 cells were resuspended at 1 × 10^7^ viable cells/mL in 50% PBS:50% Matrigel matrix (Corning), and T3M-4 cells were resuspended at 1 × 10^7^ viable cells/mL in 75% PBS:25% Matrigel Matrix (Corning); 100 µL was injected subcutaneously into the dorsal flank. MT373 (PMV Pharmaceuticals) cells were resuspended at 5 × 10^7^ viable cells/mL in 50% PBS:50% Matrigel Matrix (Corning), and 200 µL was injected subcutaneously into the dorsal flank. Mice were selected and randomized into treatment groups according to tumor size (human cell line xenografts) or 6 days after implantation (mouse syngeneic models).

Hupki-Y220C mice were generated by Cyagen Biosciences. The genomic fragment containing mouse *Trp53* exons 4 to 9 was replaced with the corresponding human genomic DNA fragment containing human *TP53* exons 4 to 9, and the Y220C mutation (*TP53* c.659A>G, p.Y20C) was introduced, along with a polymorphic codon 72 that encodes arginine instead of proline (*TP53* c. 215C>G, p. P72R).

### *In Vivo* Treatments

For *in vivo* administration, PC14374 or PC14586 was formulated in either 2% hydroxypropylcellulose and 0.5% Tween 80 (v/w in water), or 2% hydroxypropylcellulose, respectively. PC14586 or PC14374 was orally administered (gavage) at the indicated doses; 200 µg of anti–PD-1 (Bio X Cell, Cat. # BE0146, RRID: AB_10949053) was administered intraperitoneally every 3 days for the length of the study. Mice in groups with CD8^+^ depletion received 250 µg of CD8^+^ (Bio X Cell, Cat. # BE0061, RRID: AB_1125541) intraperitoneally starting 3 days before the start of the study and then twice weekly for the length of the study.

### Tumor Protein Sample Preparation

Lysis buffer (Cell Signaling Technology, Cat. # 9803) was added to tumor samples and homogenized using a TissueLyser LT (Qiagen) per the manufacturer’s instructions. Homogenized samples were centrifuged for 30 minutes at 20,817 × *g* at 4°C, and the supernatant was transferred to a 1.5 mL tube. Protein samples were quantified using bicinchoninic acid assay according to the manufacturer’s instructions (Thermo Fisher Scientific).

### ELISA

The 96-well ELISA plates were coated with WT p53 (150 ng/well; PAb1620; Caprico Biotechnologies, Cat. # 102201, RRID: AB_3662135), mutant p53 (100 ng/well; PAb240; Novus Biologicals, Cat. # NB200-103, RRID: AB_10001083), or total p53 (3.13 ng/well; PAb1801; Novus Biologicals, Cat. # NB200-104, RRID: AB_10001307) antibodies and incubated overnight at 4°C. Plates were washed with wash buffer (PBS + 0.05% Tween 20) and treated with blocking buffer (PBS + 1% BSA + 0.05% Tween 20) for 1 hour and then washed. Tumor lysates were diluted in blocking buffer to the specified protein amount up to a 100 µL volume (WT p53, 50 µg; mutant p53, 12.5 µg; total p53, 5 µg). Lysates were incubated overnight at 4°C with shaking. Plates were washed and treated with detection antibody diluted in blocking buffer (0.025 mg/mL; biotinylated p53; Cell Signaling Technology, Cat. # 4667, RRID: AB_10693641) for 1 hour. Then plates were washed and finally incubated in streptavidin–horseradish peroxidase (HRP) (1:10,000; Pierce) diluted in blocking buffer for 30 minutes. Plates were washed, and the reaction was developed using 3,3ʹ, 5,5″-tetramethylbenzidine (Cell Signaling Technology) for ≈5 minutes. The reaction was quenched with 0.16 mol/L of sulfuric acid. Plates were read on a plate reader at 450 nm (SpectraMax Plus, Molecular Devices, RRID: SCR_018598). A background measurement was subtracted from the treated samples, and they were normalized to their respective vehicle controls.

The p21, MDM2, and MIC-1 ELISAs were performed according to the manufacturer’s instructions (R&D Systems, Cat. # DYC1047, DYC1244-2, and DY957). In brief, 96-well plates were coated with the respective capture antibody and incubated overnight at 4°C. Plates were then washed in wash buffer and blocked for 1 hour. Tumor lysates (12.5 µg p21, 75 µg MDM2, or 25 µg MIC-1) or plasma (MIC-1) was diluted to the appropriate concentration and added in a volume of 100 µL. Additionally, a seven-point standard curve was added to the plates. Plates were incubated for 2 hours at RT (p21, MIC-1 plasma) or 4°C overnight (MDM2 protein) with shaking. The plates were then washed and incubated in detection antibody for 2 hours. Plates were washed and incubated in streptavidin–horseradish peroxidase for 30 minutes. Finally, plates were washed, and the reaction was developed using 3,3ʹ, 5,5″-tetramethylbenzidine substrate for 10 minutes. The reaction was quenched with stop solution (0.16 mol/L H_2_SO_4_), and the plates were read at 450 and 570 nm (SpectraMax Plus, Molecular Devices). Protein levels for both analytes were quantified using the provided standard curve.

### MSD

As per the Meso Scale Discovery (MSD) U-Plex manufacturer’s protocol, mutant p53 (PAb 240; Novus Biologicals, Cat. # NB200-103, RRID: AB_10001083, 5 µg/mL), WT p53 (PAb1620; Caprico Biotechnologies, Cat. # 102201, RRID: AB_3662135, 10 µg/mL), total p53 (PAb1801; Novus Biologicals, Cat. # NB200-104, RRID: AB_10001307, 5 µg/mL), and p21 Waf1/Cip1 (DCS60; Cell Signaling Technology, Cat. # 2946, RRID: AB_2260325, 0.5 µg/mL) antibodies were coupled with U-PLEX linkers by combining optimized concentrations for each antibody with the assigned linker and then vortexed and incubated for 30 minutes at RT before adding stop solution and incubating for another 30 minutes. The coupled antibody–linkers were combined into the same tube, and the total volume was made up with stop solution. The 96-well MSD U-PLEX plates were coated with 50 µL/well of combined antibody–linker solution and incubated overnight at 4°C on a shaker. Plates were washed three times with wash buffer (1× TBS + 0.1% Tween 20) and blocked with 1× blocking buffer (1× TBS + 0.1% Tween 20 + 3% BSA). Tumor lysates were diluted in 1× lysis buffer to 0.4 µg/µL. After the blocking buffer was aspirated from the MSD plate, 50 µL of tumor lysate was added to each well, and the plates were incubated overnight at 4°C on a shaker. Plates were washed three times and treated with 50 µL/well detection antibody diluted in antibody diluent buffer [1 × TBS + 0.1% Tween 20 + 1% BSA; 0.05 µg/mL p53 7F5 rabbit mAb (Cell Signaling Technology, Cat. # 2527, RRID: AB_10695803); and 0.05 µg/mL p21 12D1 rabbit mAb (Cell Signaling Technology, Cat. # 2947, RRID: AB_823586)] for 1 hour at RT. The plate was washed three times, and the secondary antibody (goat anti-rabbit SULFO-TAG at 1 µg/mL) was added at 50 µL/well and incubated for 1 hour at RT on a shaker. The plate was washed three times, 2× MSD read buffer was added at 150 µL/well, and the plate was analyzed on a MESO QuickPlex SQ 120 (Meso Scale Diagnostics, Cat. # 3-1300-0B0002-F, RRID: SCR_020304).

### Cycloheximide Treatment

NUGC-3 cells were pretreated with cycloheximide (100 µg/mL, Sigma-Aldrich, Cat. # C7698) to inhibit protein synthesis. NUGC-3 cells were then treated with PC14374 or PC14586 for 4 hours. Cells were harvested and analyzed using the p53 conversion ELISA.

### Cellular Oligonucleotide Pull-Down

Biotinylated oligonucleotides (DNA duplexes with 5ʹ to 3ʹ forward sequences and biotin attached to the 3ʹ end of the complementary DNA sequences) were bound to MSD U-PLEX plates according to the manufacturer’s protocol overnight with agitation at 4°C. The forward sequences are as follows: ATTAGGCATGTCTAGGCATGTCTAGG (for consensus), ATTACTGTGCAGTACTGTGCAGTAGG (for scramble), AGGAACATGTCCCAACATGTTGAG [for CDKN1A (p21)], GCAGAACATGTCTAAGCATGCTGGGC (for GADD45A), and CGCCTGCAAGTCCTGACTTGTCCGCG [for BBC3 (PUMA)].

The next day, plates were washed, blocked, and incubated with reactivator compound-treated cell lysates (with equal protein loading) under agitation at 4°C for 2 hours. Plates were then washed, p53 detection antibody [p53 (7F5)] was added, and the plates were incubated at 4°C overnight with agitation. On the next day, plates were washed, incubated with sulfo-tagged secondary rabbit antibody (Meso Scale Diagnostics, Cat. # R32AC-1, RRID: AB_2783819) for 1 hour at RT with agitation, and washed and developed with MSD read buffer. The signal from each oligonucleotide for each compound treatment was normalized to each DMSO control and graphed using GraphPad Prism. For cell lysate preparation, NUGC-3 cells were treated and plated in six-well plates and allowed to adhere overnight. Cells were treated with PC14586 for 2 hours and then washed in PBS and lysed in lysis buffer (Cell Signaling Technology). Protein was quantified using a bicinchoninic acid assay (Thermo Fisher Scientific), and equal protein amounts were loaded onto the MSD U-PLEX plates.

### IHC

Tumors were fixed in 10% buffered formalin overnight and then transferred to 70% ethanol for paraffin-embedding processing. Formalin-fixed, paraffin-embedded blocks were sectioned at 4-μm thickness, and sections were baked onto the slides at 60°C for 30 minutes. Predetermined IHC protocols using the Leica BOND RX autostainer were applied for p21, MDM2, and cleaved caspase-3, along with isotype control at Crown Bioscience (Supplementary Table S3). For terminal deoxynucleotidyl transferase–mediated dUTP nick end labeling (TUNEL) analysis, slides were deparaffinized, rehydrated, and then pretreated with proteinase K; equilibration buffer, terminal deoxynucleotidyl transferase, and antidigoxigenin conjugate were applied before 4′,6-diamidino-2-phenylindole staining. Images were captured using the NanoZoomer digital slide system (Hamamatsu, NDP2.0-HT). Representative images were analyzed with the HALO platform, in which necrotic and stromal areas were excluded.

### Flow Cytometry

Tumors from mice were collected and sent to Crown Bioscience in MACS Tissue Storage Solution. Tumors were subsequently digested with gentleMACS, and cell suspensions were counted. For intracellular staining, single-cell suspensions were surface-stained, fixed, and permeabilized with FoxP3 Staining Buffer (eBioscience/Thermo Fisher Scientific, Cat. # 12-5773-82, RRID: AB_465936). For surface staining, Fc blocking buffer was added to samples and washed. Samples were processed with selected antibodies on the LSRFortessa X-20 FACS machine (BD Biosciences; Supplementary Table S4). Data were analyzed by Kaluza (Beckman Coulter).

### NanoString

Total RNA from tumors was extracted using the RNeasy Mini kit (Qiagen). NanoString analysis of the isolated RNA was completed with the IO 360 profiling panel with supplemental p53 target genes (NanoString Technologies, RRID: SCR_023912) and run by NanoString Technologies. Raw data files are shown as Supplementary File S15 and were analyzed using the nSolver analysis software (NanoString Technologies, RRID: SCR_003420), with the mRNA counts normalized to housekeeping genes and the vehicle samples used as a reference.

### Cell RNA Lysates and RNA Preparation

Cells were plated in 180 µL of complete media per well in 96-well plates (or in 76 µL per well in 384-well plates) and incubated overnight as indicated. Cells were treated with 20 µL per well of 10× final concentrations of compounds (or 4 µL per well of 20× in 384-well plates dispensed by the JANUS automated workstation) for the indicated time durations. Cell RNA lysates were prepared as soon as possible to avoid any further exposure to room temperature as those carrying the Y220C-mutant p53 protein are temperature-sensitive. Briefly, the medium from the cells was quickly aspirated by the BlueWasher using “GentleSpin” evacuation. The cells were then washed with 100 µL of FastLane Cell Probe Kit wash buffer per well (or 30 µL for 384-well plates dispensed by the JANUS automated workstation). The buffer was aspirated by the BlueWasher using “GentleSpin” evacuation, and then the cells were immediately lysed using FastLane lysis buffer along with gDNA Wipeout Buffer 2 from the FastLane Cell Probe Kit, per the manufacturer’s instructions, at 40 to 50 µL (or 15 µL for 384-well plates) per well. RNA lysates in plates were directly heated to 80°C for 5 minutes before being diluted and immediately measured by qRT-PCR or stored at −80°C for later analysis.

The total cellular RNA was purified from cells cultured in six-well plates. Briefly, the medium was quickly aspirated after the plate was removed from the incubator, and then the cells were immediately lysed in 350 µL of Buffer RLT (Qiagen) supplemented with 10 µL/mL of β-mercaptoethanol to avoid any further exposure to room temperature. RNA was purified either manually by using the RNeasy Mini Kit with DNase I digestion or automatically by QIAcube with DNase I digestion, following the manufacturer’s protocol “Purification of Total RNA from Animal Tissues and Cells Including DNase Digestion.”

Total tumor RNA was purified from liquid nitrogen flash-frozen tumor samples. Briefly, frozen tumor samples were cut on dry ice to <15 mg and lysed in Buffer RLT (Qiagen) with 10 μL/mL of β-mercaptoethanol using the TissueLyser LT (Qiagen), following the manufacturer’s protocol “Purification of RNA or Multiple Analytes from Animal and Human Tissues.” Total RNA was further purified from the lysates by QIAcube (Qiagen) with DNase I digestion, using the manufacturer’s protocol “Purification of Total RNA from Animal Tissues and Cells Including DNase Digestion.” RNA concentration was measured using a NanoDrop 2000 spectrophotometer.

### qRT-PCR

Individual gene expression analysis was carried out using one-step TaqMan-based qRT-PCR. For cell RNA lysates in FastLane lysis buffer, each was diluted 10 to 20 times in RNase-free water before using 4 µL each in the qRT-PCR assay, 20 µL reaction, using the LightCycler 96 or LightCycler 480. For the purified RNA from cells or tumor samples, each was diluted to 2.5 ng/μL in DNase- and RNase-free water, and 10 ng was used for each qRT-PCR assay in a 20 μL reaction using the LightCycler 96. In each of the above assays, the QuantiTect Probe RT-PCR Kit was used along with the specific TaqMan primer/probe sets as indicated in individual channels, following the manufacturers’ instructions. The expression of a gene of interest (e.g., p21) relative to the reference gene GAPDH in a ratio to the DMSO control was calculated by the ΔΔCt method. As the LightCycler 96 and LightCycler 480 use different algorithms to calculate Ct (Cq or Cp), the basal levels of the relative expression (e.g., p21/GAPDH) were slightly different, resulting in varying fold changes for the same test. Qualitatively, expression changes remained the same.

### p53 Pathway Gene Profiling

The p53 signaling pathway profiling was done using SYBR Green–based qRT-PCR after the RT. In detail, the first strand of cDNA was synthesized from 500 ng of purified total RNA from each sample using the RT^2^ First Strand Kit before being mixed with RT^2^ SYBR Green qPCR Mastermix. Subsequently, the mixture was applied to the RT^2^ Profiler PCR Array Human p53 Signaling Pathway, Plate F, and detected by the LightCycler 96, following the manufacturers’ instructions. At least three samples in each group were used for the profiling. Data were analyzed by the GeneGlobe Data Analysis Center (RRID: SCR_021211) from the Qiagen website after uploading Ct values of profiled genes from all groups of samples, using the average Ct values of five housekeeping genes on the plate as the reference control to normalize plate-to-plate variation. Additionally, a similar result was achieved by the ΔΔCt method, using five housekeeping genes as the first reference control and the DMSO control group as the second reference control.

### RNA-Seq

The total RNA purification was performed as described above. RNA samples were quantified using a Qubit 2.0 Fluorometer (Thermo Fisher Scientific, RRID: SCR_020553), and the RNA integrity was checked with a 4200 TapeStation (Agilent Technologies; RRID: SCR_018435). Samples were initially treated with TURBO DNase (Thermo Fisher Scientific) to remove DNA contaminants. rRNA depletion was performed using the FastSelect rRNA HMR Kit (Qiagen, Cat. # 335376), and strand-specific RNA-seq libraries were prepared using the NEBNext Ultra II Directional RNA Library Prep Kit for Illumina (New England Biolabs, Cat. # E7760L), following the manufacturers’ instructions. Paired-end (2 × 150 bp) sequencing was performed using an Illumina HiSeq X Ten system (RRID: SCR_016385) at Azenta Life Sciences (RRID: SCR_003177). Paired end reads were aligned to the human genome GRCh38 using the STAR aligner (GitHub version 2.7.7a, RRID: SCR_004463; Ref. 43). Fragments were assigned to the genes (GENCODE annotation, RRID: SCR_014966) using featureCounts (GitHub version 1.4.6-p5, RRID: SCR_012919; refs. 44, 45). Differential expression analysis was performed using R [version 4.1.3; R Core Team (cited March 21, 2022). Available from: https://www.r-project.org/] package DESeq2 (Bioconductor, version 1.34.0, RRID: SCR_015687; ref. 46), testing for fold changes significantly >1.5 to identify DEGs. Fragments per kilobase of transcript per million mapped reads (FPKM) values were computed using the edgeR package (version 3.36.0) using the trimmed mean of M values normalization method. Transcripts per million values were computed from FPKM values, assuming that the abundance of each transcript is proportional to its FPKM value (47). The *P* value was adjusted using the Benjamini–Hochberg method (*P* adjusted) as the FDR *q* value. A cutoff of *q* value ≤0.05 was applied to select significant DEGs. Significant DEGs ranked by Wald stat were further analyzed with the GSEA Preranked tool in GSEA software (Broad Institute, version 4.3.2, build 13, RRID: SCR_003199; refs. 48, 49) using the gene sets from the Molecular Signatures Database C2 (curated gene sets) collection (Broad Institute, version 2023.2, RRID: SCR_016863), supplemented with one gene set of the direct p53-targeted lncRNAs, which contains 86 lncRNAs that are upregulated by p53, each having a recurrent p53-binding site close to its transcription start site (7).

### Statistical Analysis

The GeneGlobe Data Analysis Center from Qiagen was used for statistical analysis for p53 pathway gene profiling. Statistical analyses for RNA-seq data were described above using analysis-specific software. Prism software (GraphPad Software) was used for statistical analysis for all other *in vivo* experiments. One-way ANOVA with Dunnett’s correction was used for multiple comparisons. Significance is listed in the figure legends as indicated.

### Data Availability

The datasets generated and/or analyzed during the current study are available from the sponsor/corresponding author upon reasonable request. The RNA sequence data generated in this study have been deposited in NCBI’s Gene Expression Omnibus (RRID: SCR_005012; ref. 50) and are accessible through Gene Expression Omnibus Series accession numbers GSE276223 and GSE276224.

## Supplementary Material

Supplementary Tables S1-S4 Supplementary Table S1. Sensitivity of Various Cell Lines to Reactivator Compound PC14586. Supplementary Table S2. Cell Line Information for Cells Used in the Paper. Supplementary Table S3. Antibodies Used to Detect Specific Antigens in Tumor Sections. Supplementary Table S4. Antibodies Used to Detect Specific Proteins in Tumor Samples.

Supplementary Figures S1-S7 Supplementary Figure S1. Functional Characterization of p53-Y220C Reactivator Compounds. Supplementary Figure S2. Reactivator Compounds are Active Only in p53-Y220C Cell Lines. Supplementary Figure S3. Selective Restoration of Wild-Type (WT) p53 Transcriptional Responses by PC14586 in Cells. Supplementary Figure S4. In Vivo Administration of PC14586 Leads to Tumor Growth Inhibition of Activation of Downstream Biomarkers. Supplementary Figure S5. PC14586 Restored Multiple Facets of Dynamic Wild-Type (WT) p53 Transcriptional Responses in Vivo. Supplementary Figure S6. PC14586 Administration in Vivo Led to Regulation of Genes Specific for Immune and Inflammatory Responses. Supplementary Figure S7. Clinical Timelines of Two Patients with Advanced Solid Tumors Harboring a TP53 Y220C Mutation Receiving Rezatapopt.

Supplementary File S1 p53 pathway profiling analysis in NUGC-3 cells treated by PC14586 (5 µM) vs. DMSO control (16 h).

Supplementary File S2 p53 pathway profiling analysis in NUGC-3 KO_p53i cells induced with doxycycline (50 ng/ml) vs. PBS control (12.5 h).

Supplementary File S3 p53 pathway profiling analysis in NUGC-3 KO cells treated by PC14586 (5 µM) vs. DMSO control (16 h).

Supplementary File S4 p53 pathway profiling analysis in NUGC-3 cells treated by PC14586 (5 µM) vs. DMSO control (5 h).

Supplementary File S5 p53 pathway profiling analysis in T3M-4 cells treated by PC14586 (5 µM) vs. DMSO control (5 h).

Supplementary File S6 p53 pathway profiling analysis in T3M-4 cells treated by PC14586 (5 µM) vs. DMSO control (16 h).

Supplementary File S7 p53 pathway profiling analysis in NUGC-3 KO cells treated by PC14586 (5 µM) vs. DMSO control (5 h).

Supplementary File S8 p53 pathway profiling analysis in SJSA-1 cells treated by PC14586 (5 µM) vs. DMSO control (5 h).

Supplementary File S9 p53 pathway profiling analysis in SJSA-1 cells treated by PC14586 (5 µM) vs. DMSO control (16 h).

Supplementary File S10 Annotated gene differential expression (DE) analysis from RNA-seq data in 4 cell lines (NUGC-3, T3M-4, NUGC-3 KO, and SJSA-1) treated with rezatapopt (PC14586, 5 µM) versus DMSO (5 and 16 h).

Supplementary File S11 The restoration of WT p53 transcriptional responses by PC14586 in p53-Y220C–expressing cells (NUGC-3 and T3M-4) at 5 and 16 h was assessed through GSEA analysis for DEGs.

Supplementary File S12 The restoration of the p53 signaling pathway in vivo was assessed via p53 pathway profiling analysis in NUGC-3 xenografted tumors after 6 days of 100-mg/kg PC14586 administration.

Supplementary File S13 Annotated analysis of differential gene expression (DE) from RNA-seq data in NUGC-3 xenografted tumors over 6 days of 100 mg/kg PC14586 administration.

Supplementary File S14 GSEA analysis of DEGs shows the restoration of WT p53 transcriptional responses by PC14586 in vivo in NUGC-3 xenografted tumors over 6 days of 100-mg/kg PC14586.

Supplementary File S15 The immune pathway changes with PC14374 administration in vivo based on the Nanostring data from Hupki-MT373 tumors treated with vehicle versus PC14374 at 150 or 300 mg/kg for 2Q7D×2 or 2Q7D×3.

Supplementary File S16 GSEA analysis using MSigDB Hallmark gene sets collection (H) for all detected genes demonstrated the regulation of genes for immune and inflammatory response in vivo in NUGC-3 xenograft tumors following 8 h of treatment with 100 mg/kg PC14586.
