# Supplementary material for: Restoration of the Tumor Suppressor Function of Y220C-Mutant p53 by Rezatapopt, a Small-Molecule Reactivator
Source: Cancer Discov. 2025 Feb 14;15(6):1159–79. doi: 10.1158/2159-8290.CD-24-1421 (PMC12130801; doi:10.1158/2159-8290.CD-24-1421)
Supplement: Supplementary Figures S1-S7 — Supplementary Figure S1. Functional Characterization of p53-Y220C Reactivator Compounds. Supplementary Figure S2. Reactivator Compounds are Active Only in p53-Y220C Cell Lines. Supplementary Figure S3. Selective Restoration of Wild-Type (WT) p53 Transcriptional Responses by PC14586 in Cells. Supplementary Figure S4. In Vivo Administration of PC14586 Leads to Tumor Growth Inhibition of Activation of Downstream Biomarkers. Supplementary Figure S5. PC14586 Restored Multiple Facets of Dynamic Wild-Type (WT) p53 Transcriptional Responses in Vivo. Supplementary Figure S6. PC14586 Administration in Vivo Led to Regulation of Genes Specific for Immune and Inflammatory Responses. Supplementary Figure S7. Clinical Timelines of Two Patients with Advanced Solid Tumors Harboring a TP53 Y220C Mutation Receiving Rezatapopt. [file cd-24-1421_supplementary_figures_s1-s7_suppsf1.pdf]

Supplementary Figure S1

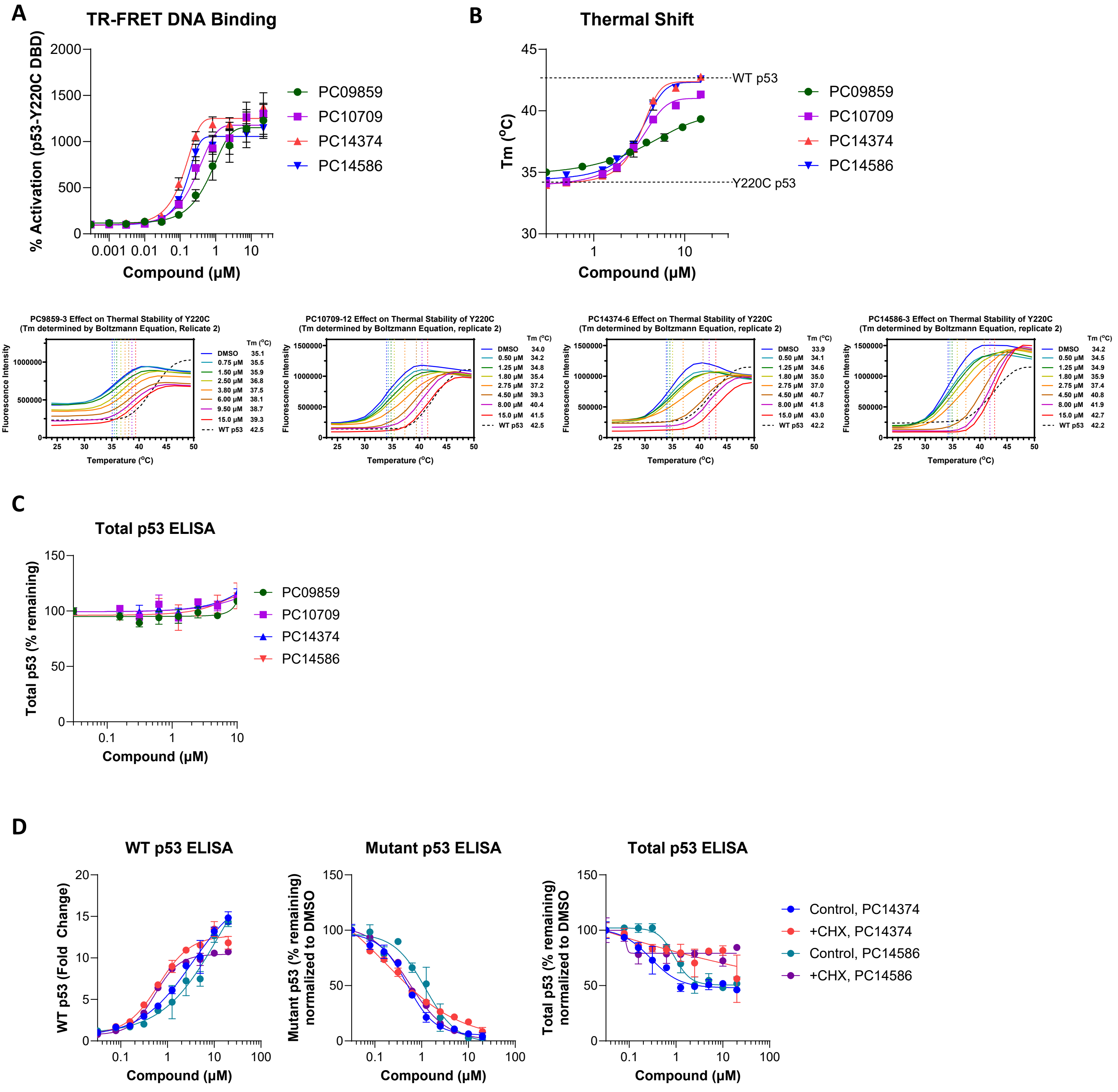

Supplementary Figure S1. Functional Characterization of p53-Y220C Reactivator Compounds. (A) Time-resolved fluorescence resonance energy transfer (TR-FRET) curves showing increased activation of Y220C-DNA binding domain (DBD) normalized to basal Y220C-DBD activity with the addition of the reactivator compounds. (B) Plots of the thermal shift assay showing an increase in melting temperature of Y220C-DBD with addition of the reactivator compounds. The dashed lines mark the melting temperatures of Y220C-DBD and wild-type (WT)-DBD. (C) Total p53 levels of NUGC-3 cells measured with total p53 ELISA after 2 h of treatment with the reactivator compounds. Data are shown as mean ± standard deviation (SD) from three experiments. (D) p53 conversion ELISA with and without cycloheximide (CHX) pretreatment (1 h) after a 4-hour treatment with PC14374 or PC14586 in NUGC-3 cells. Data are shown as the mean ± standard deviation (SD) from two experiments.

Supplementary Figure S2

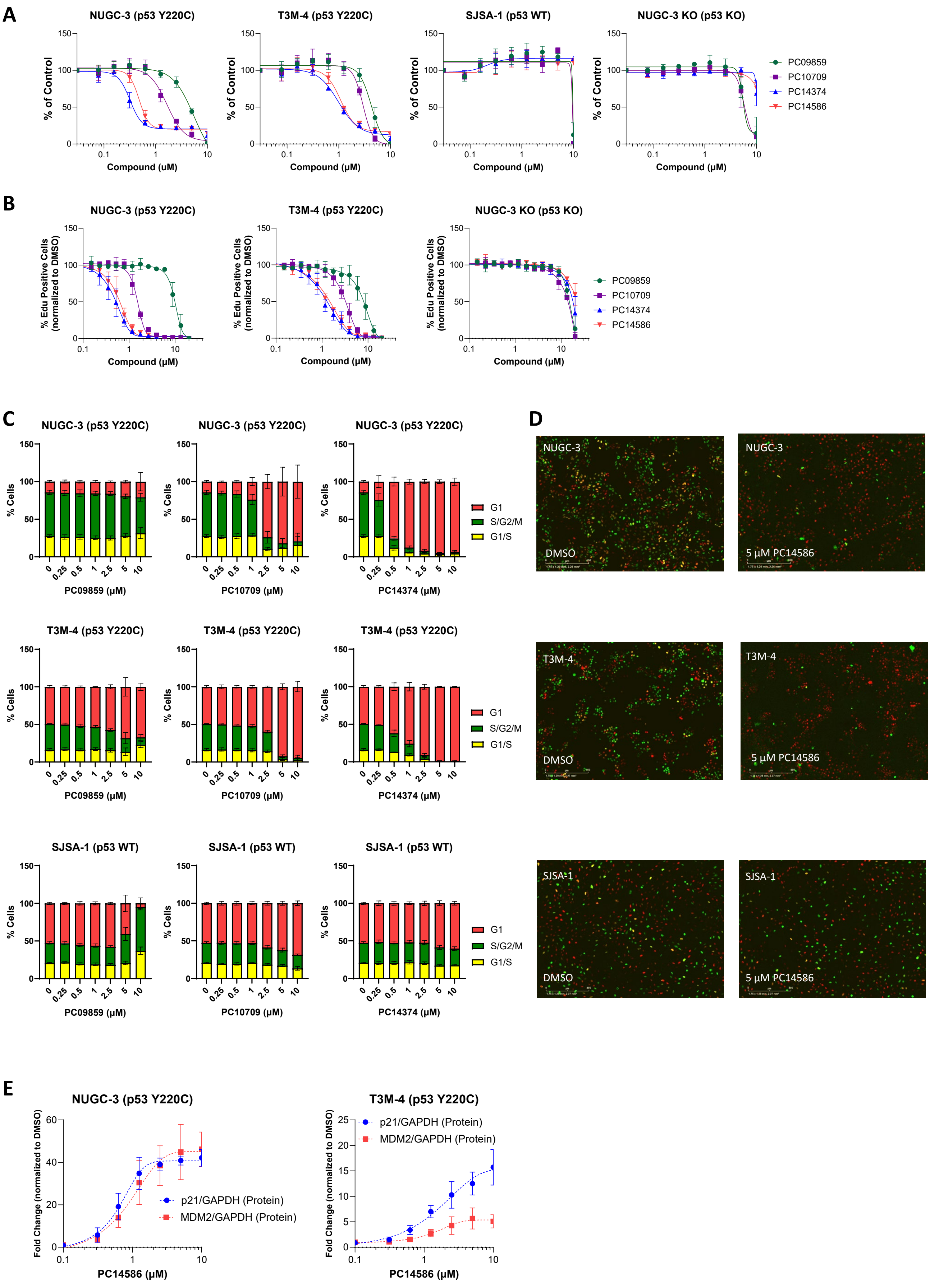

Supplementary Figure S2. Reactivator Compounds are Active Only in p53-Y220C Cell Lines.
(A) 5-Day MTT graphs of p53-Y220C compounds in four cell lines. (B) Graphs of EdU incorporation after 24 h of treatment with p53-Y220C reactivators. (C) An accumulation of cells in the G1 phase of the cell cycle in both NUGC-3 and T3M-4 cell lines after 24 h of treatment with PC10709 and PC14374, but no significant alteration with PC09859 treatment. There was no significant change seen in the SJSA-1 cell line following treatment with any of the reactivator compounds. (D) Representative images from (C) for NUGC-3, T3M-4, and SJSA-1 cell lines stably expressing the Incucyte cell cycle plasmid after 24 h of treatment with PC15486 (5μM) and a DMSO control. (E) Quantification of p21 and MDM2 Western blots following a 5-h treatment with PC14586. Data for A, B, C, and E are from three experiments and plotted as mean ± SD.

Supplementary Figure S3.

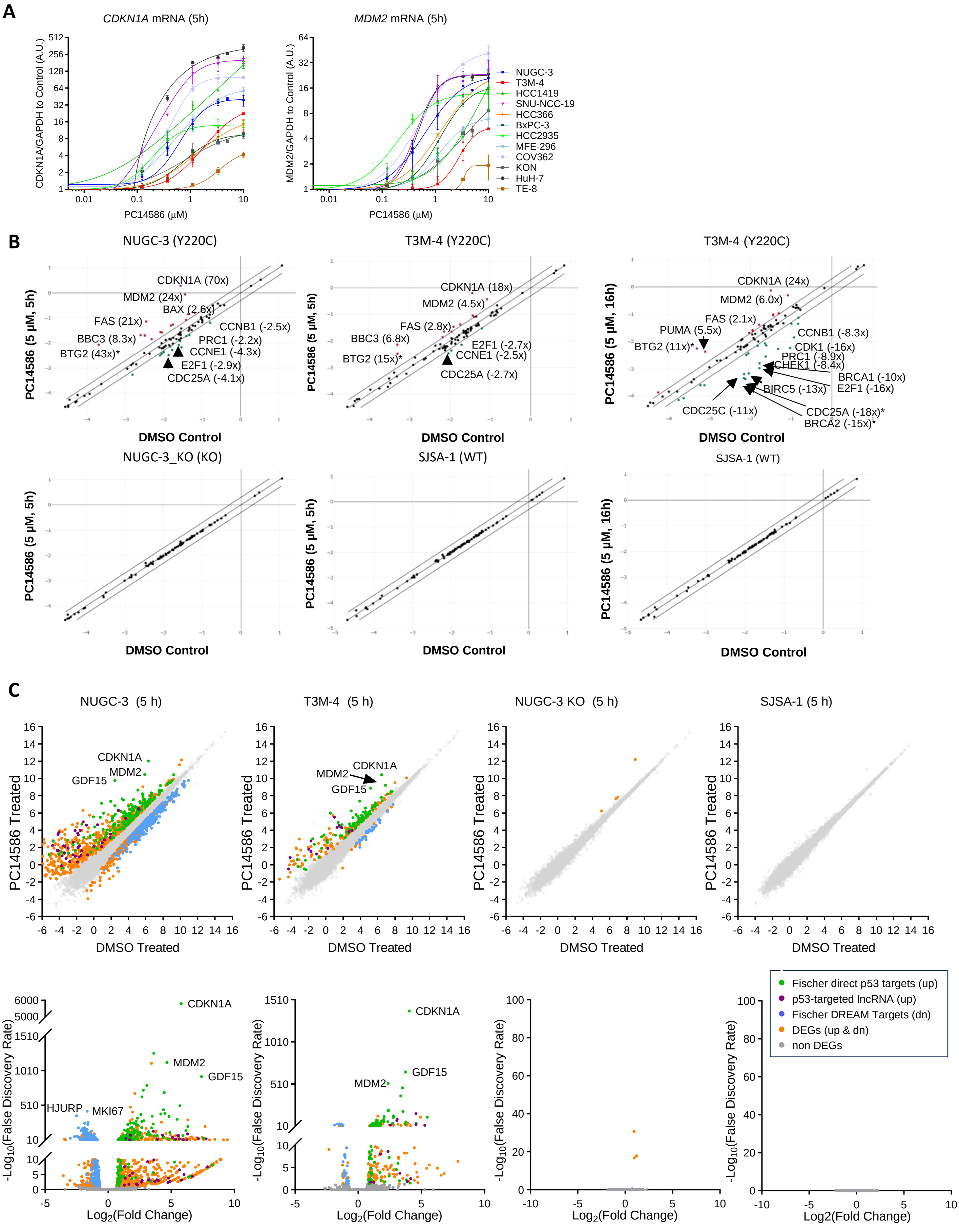

Supplementary Figure S3 (Continued)

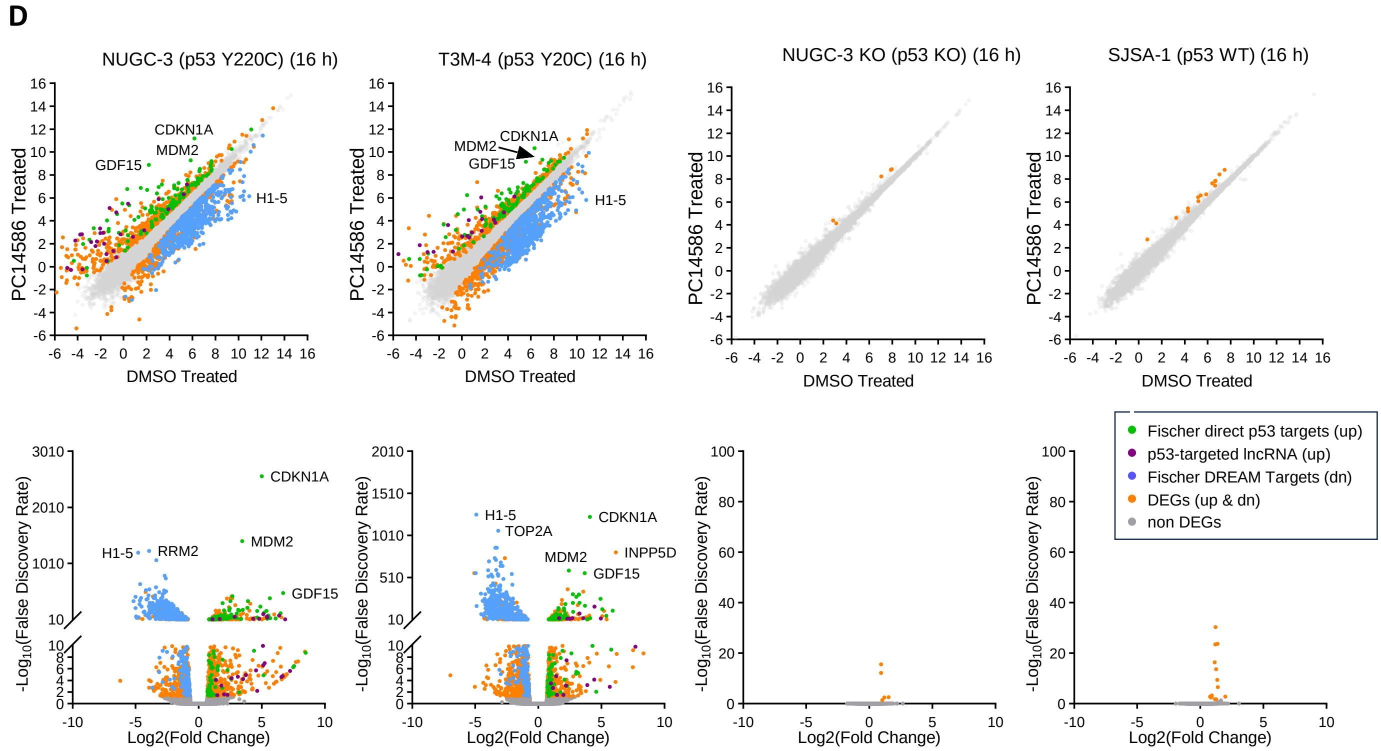

Supplementary Figure S3 (Continued)

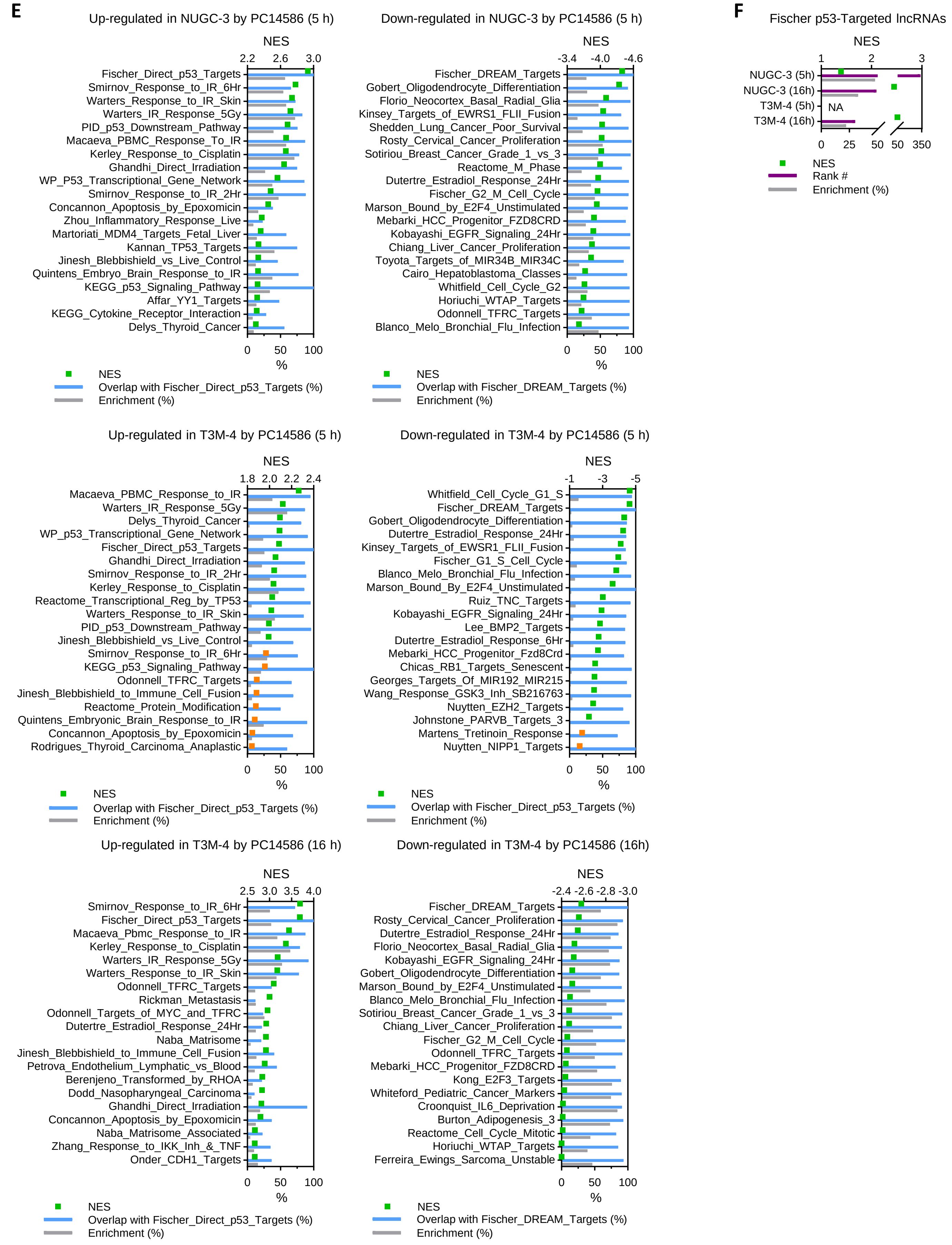

Supplementary Figure S3. Selective Restoration of Wild-Type (WT) p53 Transcriptional Responses by PC14586 in Cells.

(A) Quantification of *CDKN1A* (p21) and *MDM2* mRNA by RT-qPCR (LC480) in cell lysates from cell lines harboring p53-Y220C. See Supplementary Table S1 for cell line details. Data for NUGC-3 were representative results from four biological repeats (except the 5 µM data points from two biological repeats) and all data for other cell lines were from two biological repeats, and graphs were plotted as mean ± standard deviation. (B) Scatter plots showing expression levels of genes involved in the p53 signaling pathway in cells for 5 or 16 hours as indicated following treatment with PC14586, as performed as in Figure 2B (n=3). (C–D) Scatter and volcano plots in vitro at 5 h (C) and 16 h (d) from RNA-seq analysis of expressed genes from p53-Y220C, p53 WT and p53 knockout (KO) cell lines following DMSO and PC14586 (5 µM) treatment (16 h). Graphs were plotted as in Figures 2C and 4C. (E) Top enriched C2+ gene sets in the indicated RNA-seq data as analyzed in Figure 4D. (F) The enrichment of the supplementary Fischer p53-targeted lncRNAs gene set as described in the Methods in indicated RNA-seq data. The NES (on the upper *x* axis), rank number (#) and enrichment percentage (%) (on the lower *x* axis) were plotted.

Supplementary Figure S4

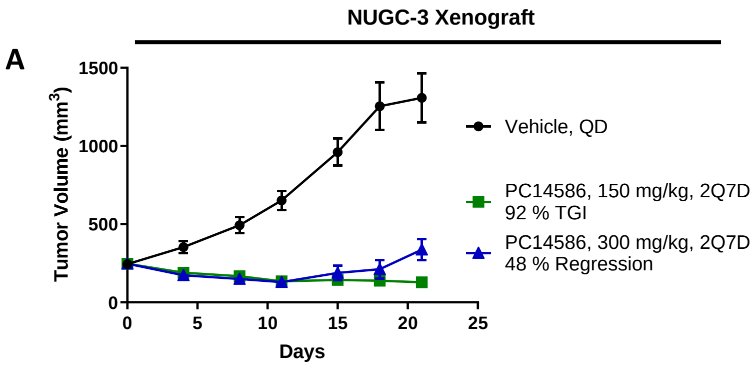

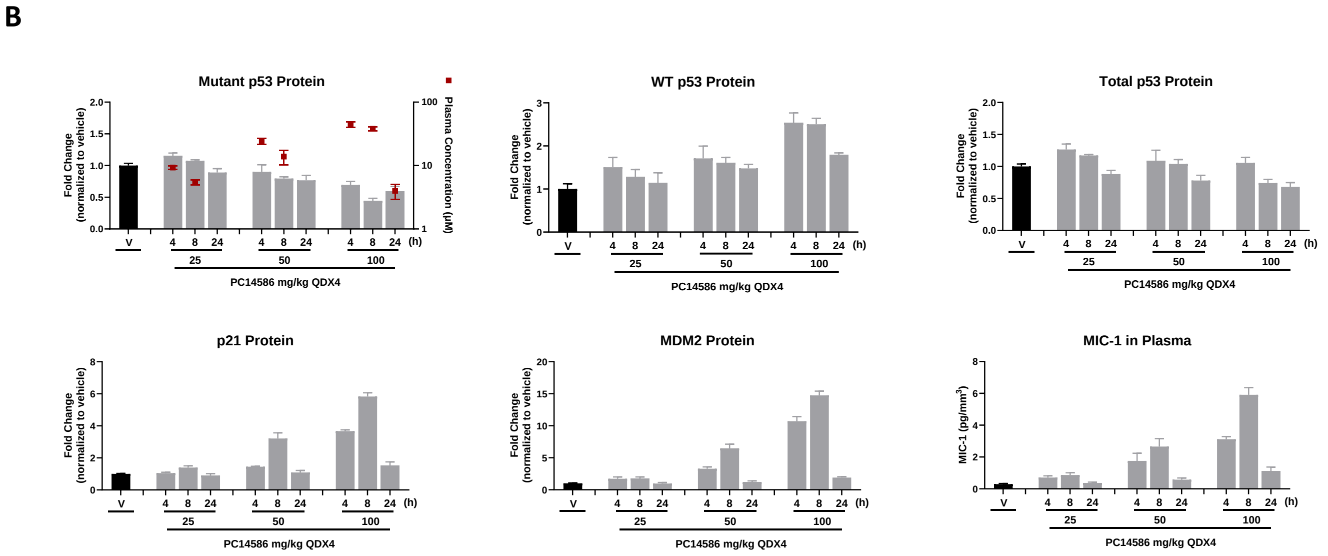

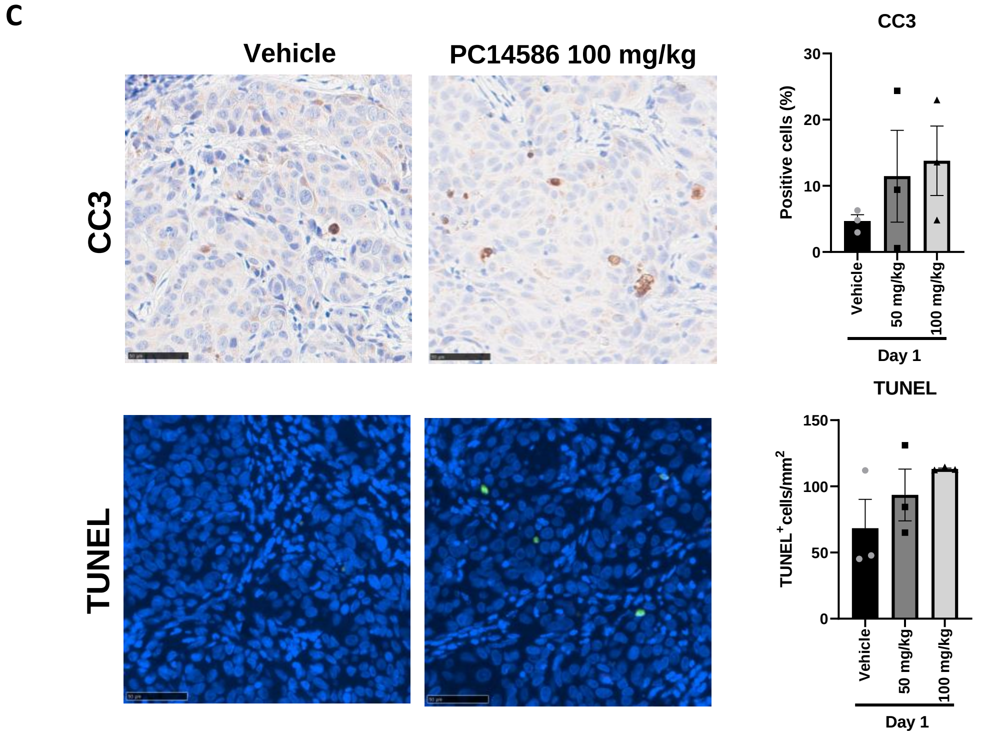

Supplementary Figure S4. In Vivo Administration of PC14586 Leads to Tumor Growth Inhibition of Activation of Downstream Biomarkers.
(A) PC14586 administration on a bolus dosing schedule led to tumor regression. In p53-Y220C–expressing NUGC-3 mouse xenografts, PC14586 or vehicle was administered orally at 150 and 300 mg/kg twice daily on the first day of a weekly cycle (2Q7D: twice daily once per week). Analysis includes tumor volume (mm^3) measurements starting on Day 1 of dosing, and each data point is the average tumor volume. Percent of tumor growth inhibition (TGI) or regression are relative to study start volume. n=10/group. Data represents mean ± standard error of the mean (SEM). (B) Evaluation of pharmacodynamic response in vivo after PC14586 administration in the T3M-4 model. Mutant, wild-type (WT), and total p53, p21, MDM2, and MIC-1 protein levels in T3M-4 xenograft tumors at the indicated time points post-dose on Day 4 of daily oral administration of vehicle, 25, 50 or 100 mg/kg PC14586. Consolidated vehicles (V) from 4, 8, and 24 h of 2QD×1 treatments, n=9; PC14586, n=4/group. Data represent mean ± SEM. Detectable plasma levels (µM) are shown by the red symbols and designated on the right *y* axis. (C) Evaluation of apoptotic events in vivo with PC14586 administration in the NUGC-3 model. Representative immunohistochemical and immunofluorescence images of CC3 and TUNEL in the NUGC-3 xenograft model after 4 days of vehicle, 50 or 100 mg/kg PC14586 daily administration. Scale bars 100 µm, 20× objective. Bar graphs represent the average percentage of positive stained cells on Day 1 following administration of 50 and 100 mg/kg PC14586. Symbols represent individual samples. Error bars ± SEM. $p > 0.1$ therefore data not significant.

Supplementary Figure S5.

A

Tumor Sample Collection Time of Mice Administered with PC14586 (100 mg/kg)

Day	Label	Administration (h)	Sample Collection (h)
1	×1	0	8, 16, 24
2	×2	0	8, 24
3	×3	0	N/A
4	×4	0	8, 24
5	×5	0	N/A
6	×6	0	8, 24

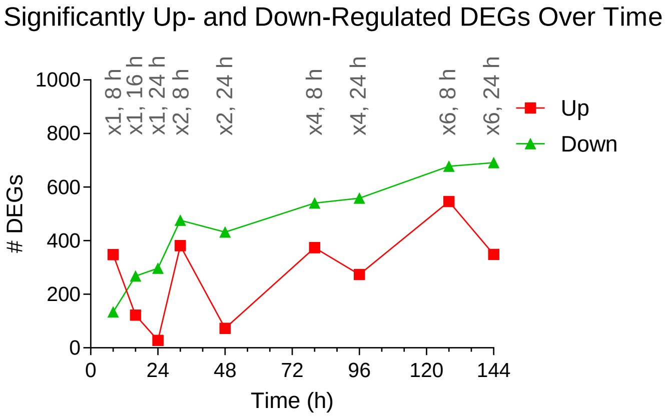

B

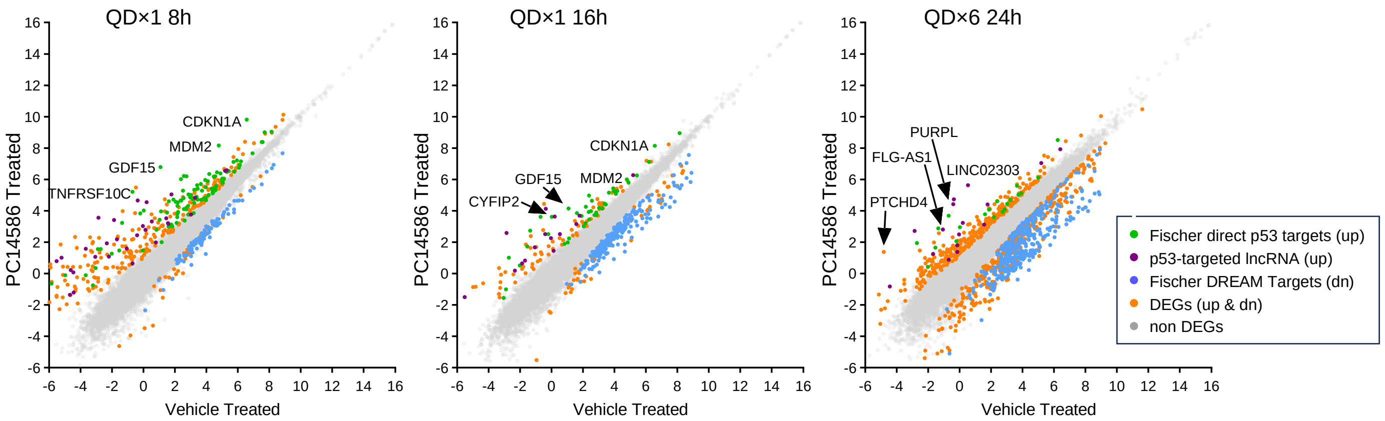

C

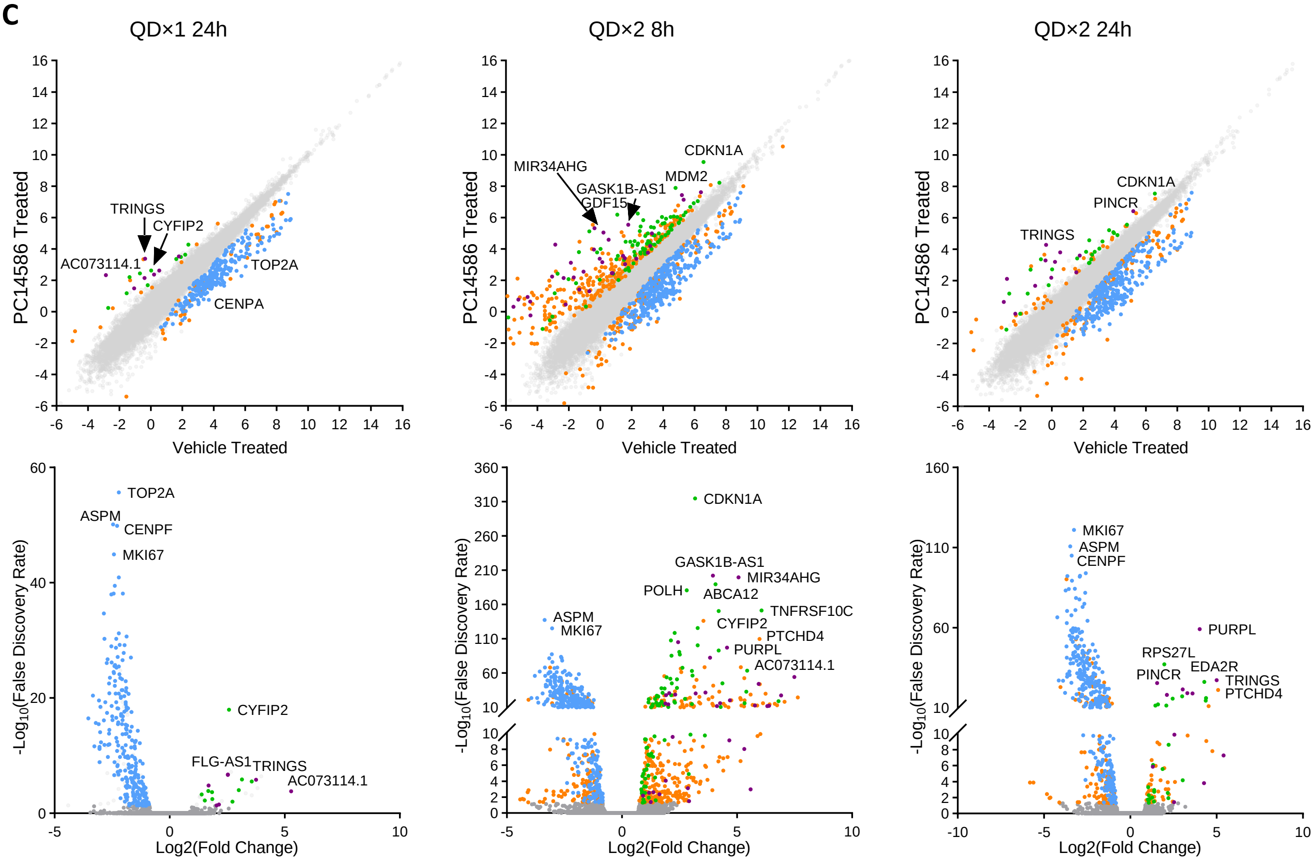

Supplementary Figure S5 (Continued)

C (Continued)

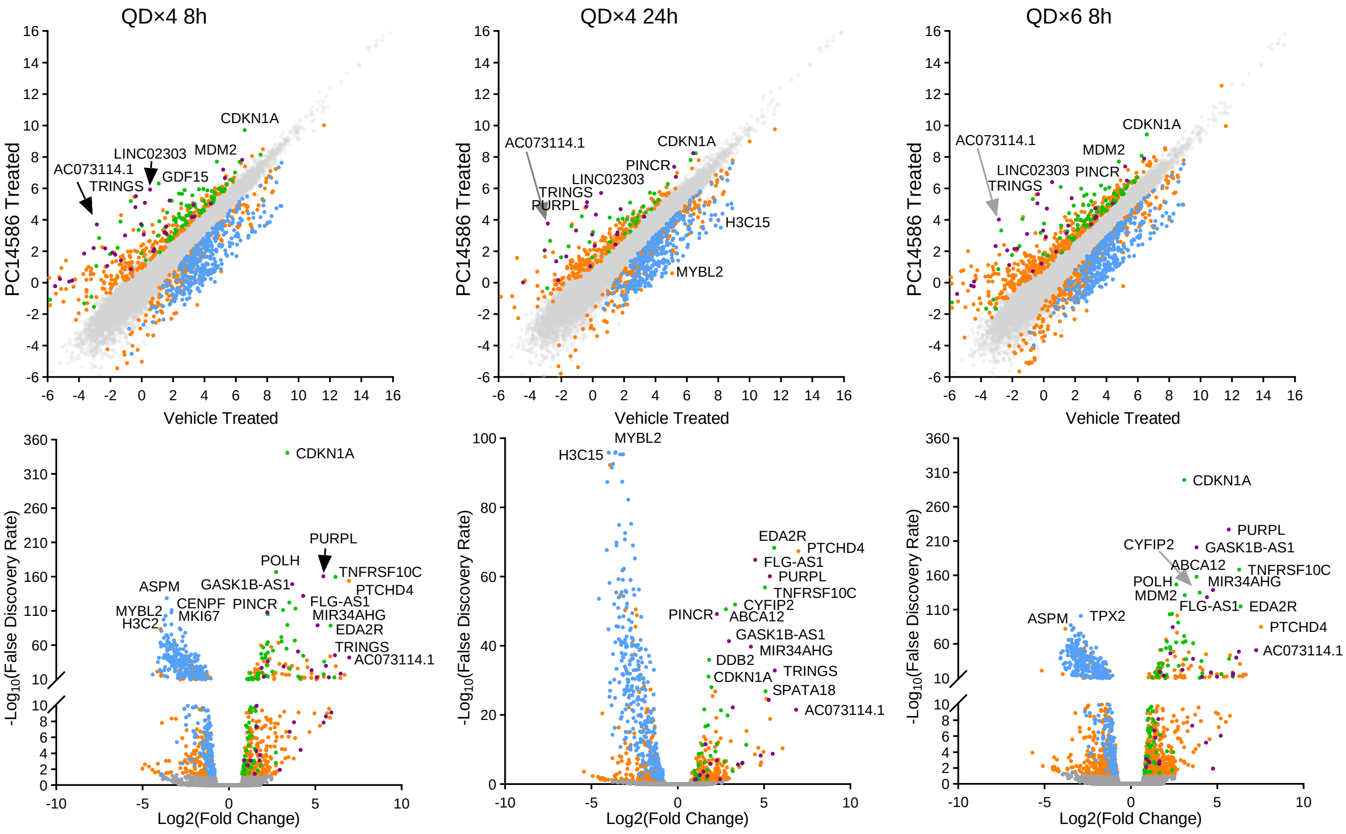

D (QD×6, 24h)

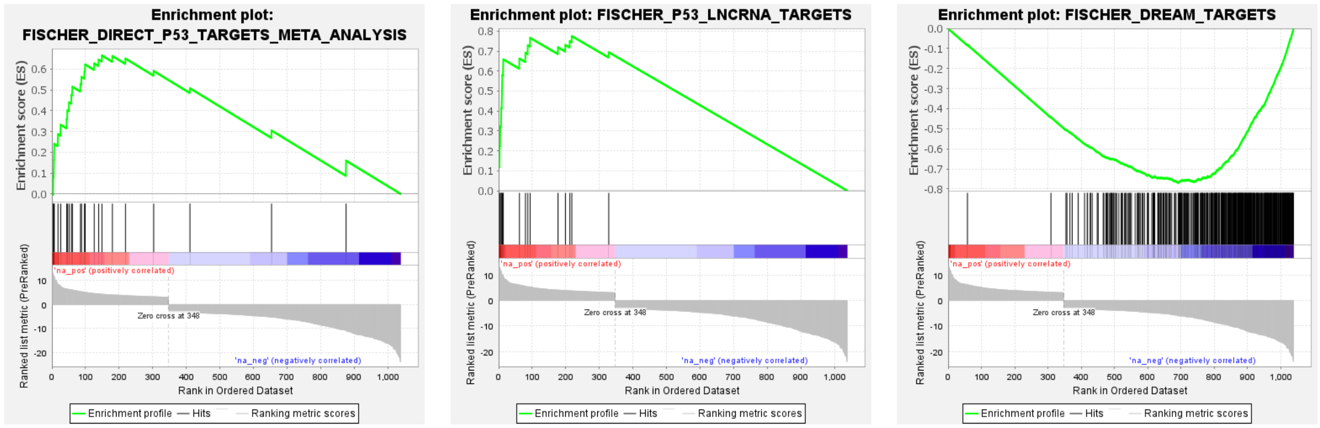

Supplementary Figure S5 (Continued)

E

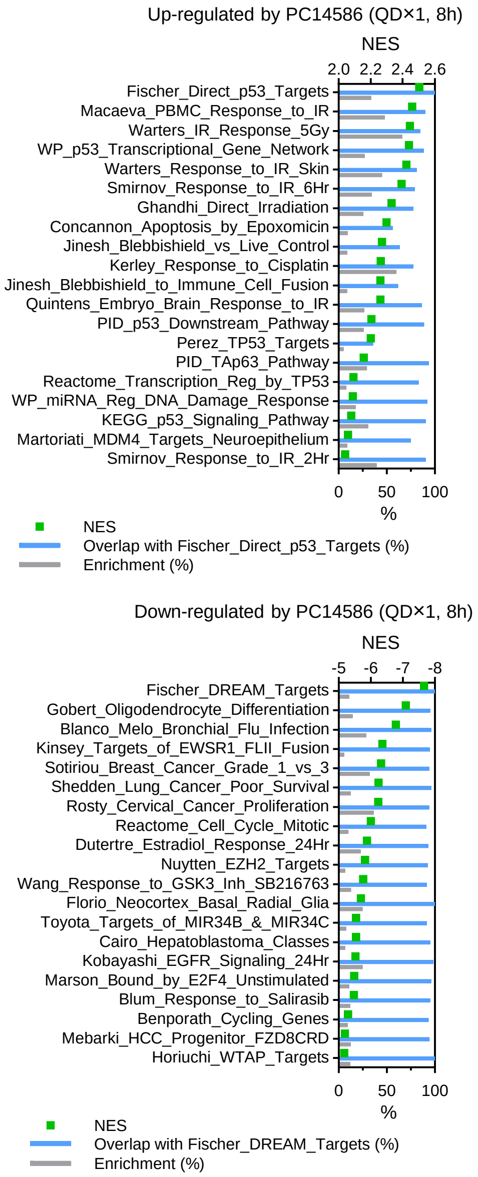

F

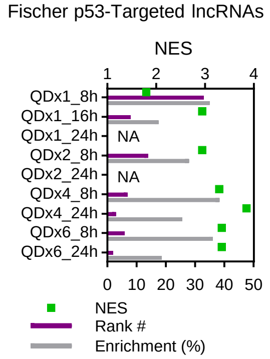

G

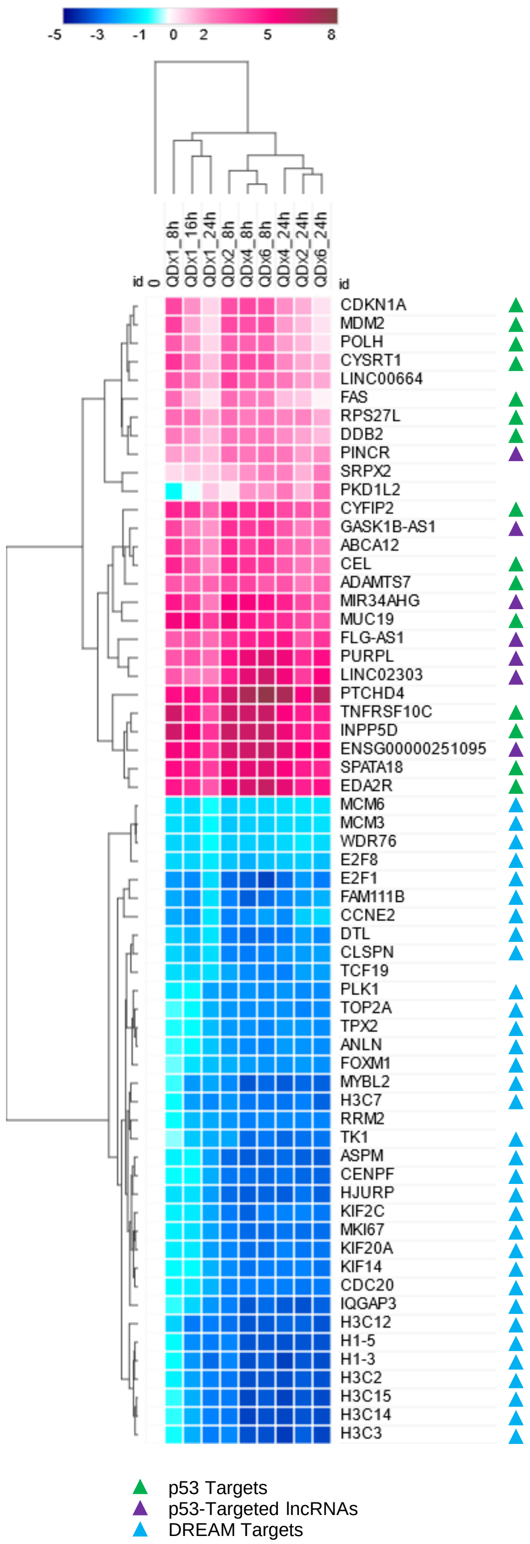

Supplementary Figure S5. PC14586 Restored Multiple Facets of Dynamic Wild-Type (WT) p53 Transcriptional Responses in Vivo.

(A) The timeline of PC14586 (100 mg/kg) administration and tumor sample collection, and the DEGs overtime. Vehicle, n=12, and PC14586, n=4 for each timepoint. (B–C) Scatter plots (B) or scatter and volcano plots (C) from RNA-seq analysis of expressed genes from NUGC-3 xenograft tumors following PC14586 (100 mg/kg) and vehicle treatment as indicated. (D) Examples of the GSEA enrichment plots from Figure 4D at 24 h. (E) Top enriched C2+ gene sets from GSEA in the indicated RNA sequencing data (PC14586 for QDx1 at 8 h) from DEGs. (F) The enrichment of the supplementary Fischer p53-targeted lncRNAs gene set in vivo analyzed as in Figure S3F (G) Heat map of top 62 genes from RNA-seq data across 6 days of 100 mg/kg PC14586 administration in vivo. The top 10 upregulated and top 10 downregulated genes from each in vivo treatment timepoint were combined and de-duplicated, and the mean value of $\log_2$ (Fold Change over vehicle) for each treatment group was graphed on the heatmap with one square per gene and per time point as indicated by the scale bar. *ENSG00000251095*, TRINGS.

Supplementary Figure S6

A

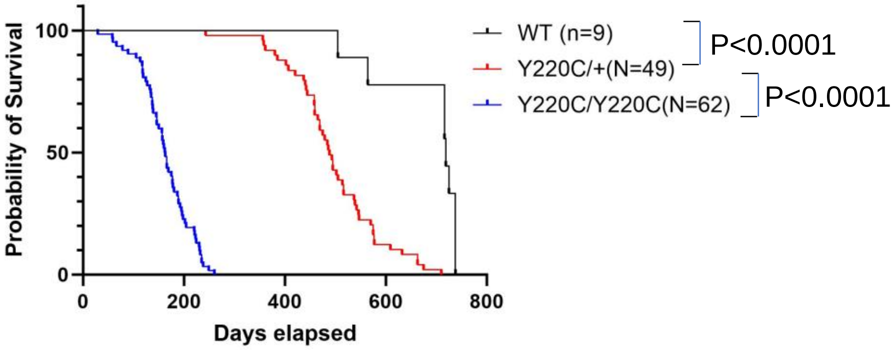

Median Survival:
WT 719 days
Y220C/+ 488 days
Y220C/Y220C 163 days

Tumor Spectrum of *Trp53Y220C* homozygous and heterozygous mice		
**Tumor Type**	*Trp53*$^{Y220C/Y220C}$ (n=44)	*Trp53*$^{Y220C/+}$ (n=35)
**Osteosarcoma**		27 (13.5%)[a]
**Sarcoma**	4	5
**Angiosarcoma**	5	3
**Histiocytic sarcoma**		1
**Spindle cell sarcoma**	1	
**Lymphoma**	36	7
**Myeloma**		1
**Squamous cell carcinoma**		1
**Malignant teratoma**	1	
**Number of tumors**	47	45
**# of mice with >1 tumor**	4	9
[a]Percent of tumors that metastasized (2 out of 27)		

B

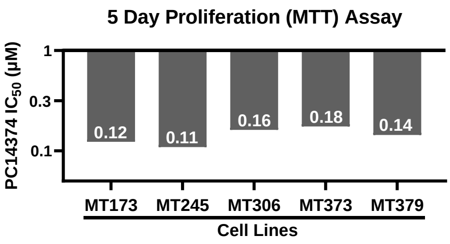

C

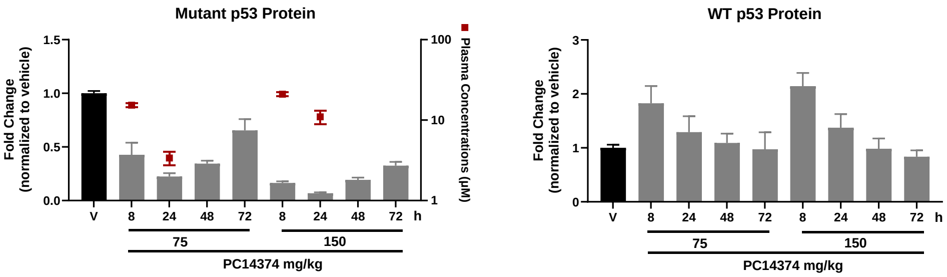

Supplementary Figure S6 (Continued)

D

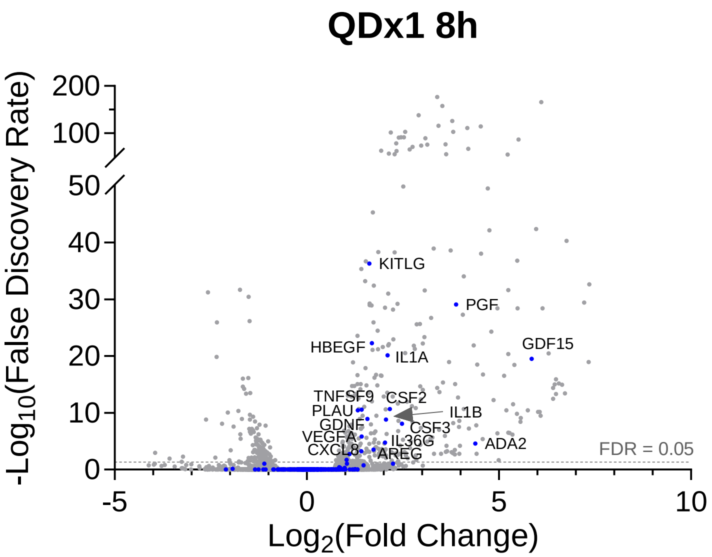

E

Regulated by PC14586 (QDx1, 8h), all genes without filtering, for GSEA with Hallmark Gene Sets

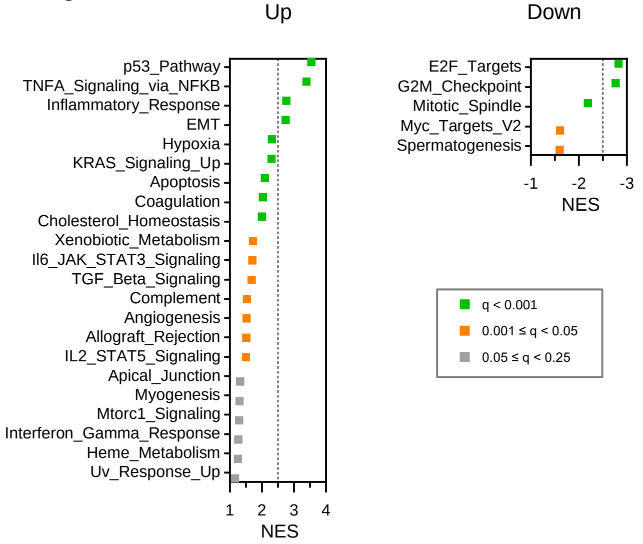

F

Top Hallmark Gene Sets (QDx1, 8h)

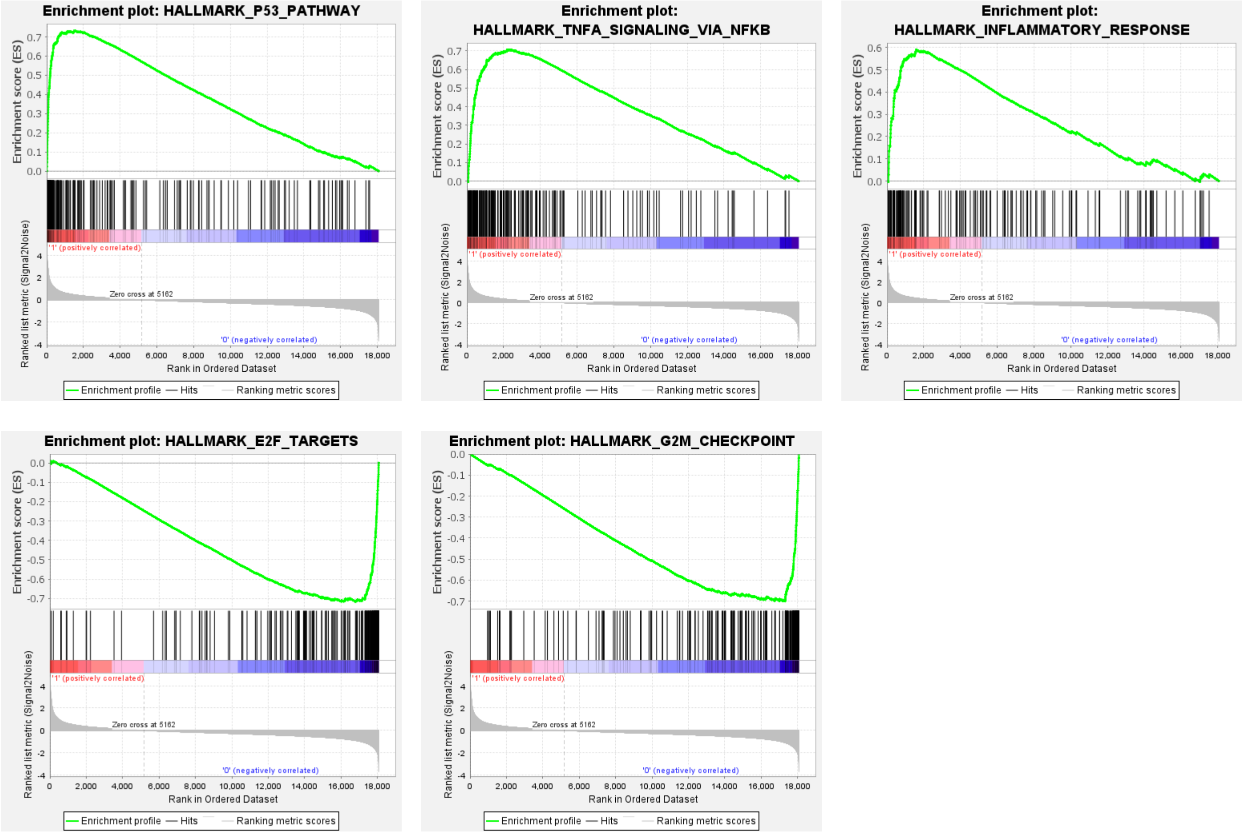

Supplementary Figure S6. PC14586 Administration in Vivo Led to Regulation of Genes Specific for Immune and Inflammatory Responses.

(A) Human Y220C p53 knock-in (i.e., HUPKI) mice were generated in a C57Bl/6 background where the human DNA binding domain (DBD; exons 4-9) containing the Y220C mutation was knocked-in to the mouse *Trp53* gene. Mice homozygous for the *TP53* Y220C mutation (C/C), show a decreased survival compared with mice heterozygous for the *TP53* Y220C mutation (C/+). Mice succumb to several tumor types, including lymphomas and sarcomas, resulting in decreased survival. Hematoxylin and eosin histology analysis by a US board-certified pathologist. Median survival time (MST) is shown. Statistical significance relative to the wild-type (WT) was determined using the Log-rank (Mantel-Cox) test (p-value: **** <0.0001). (B) A 5-day MTT analysis of cell lines generated from tumors of p53-Y220C-HUPKI homozygous mutant mice treated with PC14374 across a six-point dose response. Calculated average IC_{50} values (µM) are shown above each cell line. (C) WT and mutant p53 protein levels were detected in MT373 tumors at the indicated time points post first dose of vehicle, 75 mg/kg or 150 mg/kg PC14374 (twice daily [BID] ×1). Red symbols show detectable plasma levels. Vehicle, *n* =4/group. PC14374, *n* =4/group. Error bars show SEM. (D) Volcano plot of $-\log_{10}$ (false discovery rate) versus $\log_2$ (fold change) of genes encoding growth factors and cytokines (blue) from RNA-seq analysis of NUGC-3 xenograft tumors following PC14586 (100 mg/kg) and vehicle treatment for 8 h. Genes encoding growth factors and cytokines (blue, from the gene family of ‘cytokines and growth factors’ of GSEA MSigDB) were overlayed onto all other genes (grey). (E) Top gene sets from GSEA analysis of MSigDB Hallmark gene sets collection (H) for all genes detected without filtering in the indicated RNA sequencing data (rezatapopt [PC14586] for QD×1 at 8 h). Since the detected genes were not pre-filtered in this analysis, the NES cutoff was set to be >2.5 or <−2.5 and q to be < 0.001 as shown. See Supplementary File S16 for details. (F) Examples of the GSEA enrichment plot of Hallmark gene sets from (E).

Supplementary Figure S7.

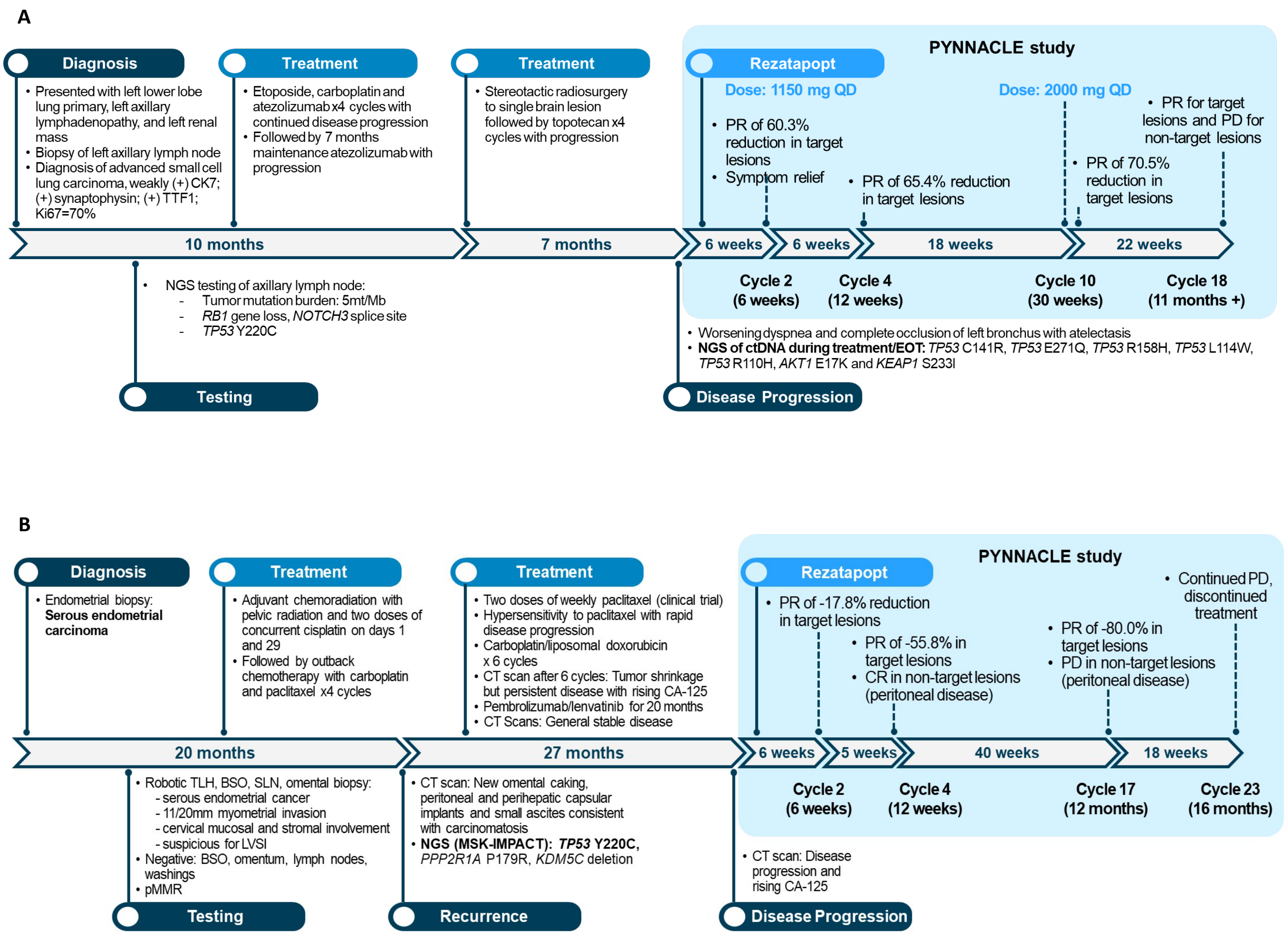

Supplementary Figure S7. Clinical Timelines of Two Patients with Advanced Solid Tumors Harboring a *TP53* Y220C Mutation Receiving Rezatapopt. (A) Clinical timeline of patient with advanced small cell lung carcinoma from the PYNNACLE Phase 1 study. (B) Clinical timeline of patient with advanced endometrial cancer from the PYNNACLE Phase 1 study. BSO, bilateral salpingo-oophorectomy; CR, complete response; CT, computed tomography; LVSI, lymph-vascular space invasion; mt/Mb, mutations per megabase; NGS, next-generation sequencing; pMMR, proficient mis-match repair; PD, progressive disease; PR, partial response; QD, once daily; SLN, sentinel lymph node; TLH, total laparoscopic hysterectomy.
